# The effects of different doses of exercise on pancreatic β-cell function in patients with newly diagnosed type 2 diabetes: study protocol for and rationale behind the “DOSE-EX” multi-arm parallel-group randomised clinical trial

**DOI:** 10.1186/s13063-021-05207-7

**Published:** 2021-04-01

**Authors:** Mark P. P. Lyngbaek, Grit E. Legaard, Sebastian L. Bennetsen, Camilla S. Feineis, Villads Rasmussen, Nana Moegelberg, Cecilie F. Brinkløv, Anette B. Nielsen, Katja S. Kofoed, Carsten A. Lauridsen, Caroline Ewertsen, Henrik E. Poulsen, Robin Christensen, Gerrit Van Hall, Kristian Karstoft, Thomas P. J. Solomon, Helga Ellingsgaard, Thomas P. Almdal, Bente K. Pedersen, Mathias Ried-Larsen

**Affiliations:** 1 Centre for Physical Activity Research, Copenhagen University Hospital - Rigshospitalet, Copenhagen, Denmark; 2 Department of Radiology, Copenhagen University Hospital - Rigshospitalet, Copenhagen, Denmark; 3 Bachelor’s Degree Programme in Radiography, Copenhagen University College, Copenhagen, Denmark; 4 Department of Clinical Pharmacology, Bispebjerg-Frederiksberg Hospital, University of Copenhagen, Copenhagen, Denmark; 5 Department of Clinical Medicine, University of Copenhagen, Copenhagen, Denmark; 6 Musculoskeletal Statistics Unit, The Parker Institute, Bispebjerg and Frederiksberg Hospital, Copenhagen, Denmark; 7 Department of Clinical Research, Research Unit of Rheumatology, University of Southern Denmark, Odense University Hospital, Odense, Denmark; 8 Biomedical Sciences, Faculty of Health & Medical Science, University of Copenhagen & Clinical Metabolomics Core Facility, Clinical Biochemistry, Rigshospitalet, Copenhagen, Denmark; 9 Blazon Scientific, London, UK; 10 Department of Endocrinology PE, Rigshospitalet, University of Copenhagen, Copenhagen, Denmark; 11 Department of Immunology & Microbiology, University of Copenhagen, Copenhagen, Denmark

**Keywords:** Randomised controlled trial, Randomised clinical trial, Type 2 diabetes mellitus, Insulin resistance, β-cell function, Lifestyle intervention, Exercise, Inflammation, Oxidative stress

## Abstract

**Background:**

Lifestyle intervention, i.e. diet and physical activity, forms the basis for care of type 2 diabetes (T2D). The current physical activity recommendation for T2D is aerobic training for 150 min/week of moderate to vigorous intensity, supplemented with resistance training 2–3 days/week, with no more than two consecutive days without physical activity. The rationale for the recommendations is based on studies showing a reduction in glycated haemoglobin (HbA1c). This reduction is supposed to be caused by increased insulin sensitivity in muscle and adipose tissue, whereas knowledge about effects on abnormalities in the liver and pancreas are scarce, with the majority of evidence stemming from in vitro and animal studies. The aim of this study is to investigate the role of the volume of exercise training as an adjunct to dietary therapy in order to improve the pancreatic β-cell function in T2D patients less than 7 years from diagnosis. The objective of this protocol for the DOSE-EX trial is to describe the scientific rationale in detail and to provide explicit information about study procedures and planned analyses.

**Methods/design:**

In a parallel-group, 4-arm assessor-blinded randomised clinical trial, 80 patients with T2D will be randomly allocated (1:1:1:1, stratified by sex) to 16 weeks in either of the following groups: (1) no intervention (CON), (2) dietary intervention (DCON), (3) dietary intervention and supervised moderate volume exercise (MED), or (4) dietary intervention and supervised high volume exercise (HED). Enrolment was initiated December 15th, 2018, and will continue until *N* = 80 or December 1st, 2021. Primary outcome is pancreatic beta-cell function assessed as change in late-phase disposition index (DI) from baseline to follow-up assessed by hyperglycaemic clamp. Secondary outcomes include measures of cardiometabolic risk factors and the effect on subsequent complications related to T2D. The study was approved by The Scientific Ethical Committee at the Capital Region of Denmark (H-18038298). Trial registration: The Effects of Different Doses of Exercise on Pancreatic β-cell Function in Patients With Newly Diagnosed Type 2 Diabetes (DOSE-EX), NCT03769883, registered 10 December 2018 https://clinicaltrials.gov/ct2/show/NCT03769883). Any modification to the protocol, study design, and changes in written participant information will be approved by The Scientific Ethical Committee at the Capital Region of Denmark before effectuation.

**Discussion:**

The data from this study will add knowledge to which volume of exercise training in combination with a dietary intervention is needed to improve β-cell function in T2D. Secondarily, our results will elucidate mechanisms of physical activity mitigating the development of micro- and macrovascular complications correlated with T2D.

**Supplementary Information:**

The online version contains supplementary material available at 10.1186/s13063-021-05207-7.

## Introduction and rationale

Historically, type 2 diabetes (T2D) has been regarded as a treatable, yet chronic, condition. Glycated haemoglobin (HbA1c) is a diagnostic tool as well as an important indicator of long-term glycaemic control with the ability to reflect the average blood glucose level during the preceding 2 to 3 months [1]. Lifestyle intervention including physical activity forms the basis of clinical care of T2D. The current exercise recommendation for T2D is aerobic exercise for 150 min/week of moderate to vigorous intensity, supplemented with resistance training 2–3 days/week, with no more than two consecutive days without physical activity [2, 3]. The rationale for the exercise recommendations for T2D relies heavily on the consistent evidence supporting the efficacy of exercise in reducing HbA1c in T2D patients [2–6] . However, evidence suggests that targeting HbA1c, i.e. the mean reduction in glucose, as the main marker for glycaemic control is not sufficient in order to minimise micro- and macrovascular complications. This is underpinned by a randomised, clinical study of 10,251 T2D patients with established cardiovascular disease or cardiovascular risk factors. The study compared standard therapy with intensive therapy targeting HbA1c and found no significant reduction in nonfatal myocardial infarction, nonfatal stroke, or death from cardiovascular causes. However, a marginal benefit was observed for microvascular complications, i.e. microalbuminuria [7]. Variables such as plasma glucose fluctuations (i.e. glycaemic variability (GV)) [8–10] have been associated with poorer cardiovascular outcome as compared to sustained hyperglycaemia. Thus, GV might be taken into account when evaluating glucose control. A resent systematic review and meta-analysis found a dose-response relationship between physical activity and all-cause mortality in patients with T2D [11]. In essence, most clinical exercise interventions targeting T2D base their conclusions on HbA1c, but to fully uncover the efficacy of exercise on T2D, we aim to look at β-cell function and other markers of diabetes pathophysiology.

### β-cell dysfunction

Although insulin resistance is the earliest detectable abnormality in T2D [12], dysfunction in the insulin secretory capacity is the major determinant of hyperglycaemia and onset of T2D [13]. By the time of T2D diagnosis, the insulin secretory capacity of the β-cell may be reduced by > 50% [14, 15]. This reduction was assessed by disposition index (DI) which is recognised as the most sensitive marker of β-cell function [15]. The detrimental effects of obesity on β-cell function are well-recognised [16, 17]. A genetic predisposition along with a chronic positive energy balance and pre-existing peripheral insulin resistance may lead to hepatic fat accumulation and subsequent hepatic insulin resistance [17, 18]. This leads to an increase in plasma glucose, which stimulates insulin secretion further enhancing the accumulation of liver fat and failure of insulin-induced suppression of hepatic gluconeogenesis.

The molecular mechanism leading to hepatic insulin resistance is suggested to be that fatty acids within hepatocytes may be oxidised for energy or are combined with glycerol to form mono-, di-, and then triacylglycerols (MAGs, DAGs, and TAGs). Excess intracellular diacylglycerol (DAG) activates protein kinase C epsilon type (PKCε) that inhibits the insulin receptor signalling pathway, thus resulting in inhibition of glycogen synthesis and activation of gluconeogenesis [16]. The chronic excess energy availability, hyperinsulinemia, and hepatic insulin resistance promote hepatic de novo *lipogenesis* that increases delivery of lipids from the liver to the circulation, tissues, and organs [19], including the pancreas where they will accumulate [17]. Due to peripheral (muscle and adipose tissue) and central (hepatic) insulin resistance, increased levels of portal insulin develop and may further stimulate hepatic de novo *lipogenesis*. This augments the storage of lipids in β-cells [17]. Intracellular lipid accumulation in the β-cells eventually leads to secretory dysfunction. This self-reinforcing cycle between the liver and the pancreas, known as the *twin cycle hypothesis* [20], may compromise β-cell insulin secretion. Consequently, the β-cell can no longer compensate for the peripheral insulin resistance in response to ingested glucose and promotes the onset of hyperglycaemia.

According to the β-cell centric hypothesis proposed by Schwartz et al. [21], the β-cell dysfunction is the sole common denominator for diabetes aetiology. The β-cell dysfunction and subsequent hyperglycaemia is the culprit for the generation of excess reactive oxygen species (ROS) and for the subsequent oxidative stress (OS). This OS induced by hyperglycaemia is suggested to be the unifying complication impetus in T2D. Thus, in supplement to HbA1c, it may be beneficial to focus on mechanisms alleviating β-cell dysfunction and subsequent vascular complications, when evaluating the significance of exercise in the clinical care of prevalent T2D [5, 6, 22, 23].

### Influence of the toxic diabetic milieu on the β-cell

The mechanism behind β-cell dysfunction may include an abundance of excess energy, consisting of fatty acids and glucose, escalating the production of ROS and causing inflammation [18, 21, 24, 25]**.** Chronic hyperglycaemia (i.e. glucotoxicity) has been shown to induce β-cell apoptosis by increasing proapoptotic gene expression while antiapoptotic gene expression remains unaffected [18]. Also, glucotoxicity increases malonyl-CoA levels, which leads to inhibition of carnitine palmitoyl transferase-1 and a subsequent decrease in fatty acid oxidation. For β-cells to secrete insulin in response to glucose, adenosine triphosphate (ATP) production must take place, but excess of fatty acids and TAGs are thought to inhibit this process. Under physiological conditions, when glucose enters the β-cell through glucose transporter 2, glucose undergoes glycolysis and the tricarboxylic acid cycle to generate ATP. However, increased fatty acid availability (i.e. lipotoxicity) inhibits both pyruvate cycling and pyruvate dehydrogenase activity, inhibiting the ATP synthesis and thereby diminishing insulin secretion [17]. Moreover, endoplasmic reticulum stress caused by gluco- and lipotoxicity may cause a depletion of Ca^2+^ stores and further prevent the release of insulin [26, 27]. Lipotoxicity activates the unfolded protein response in endoplasmic reticulum (ER) and increases both OS and transcriptional factors, e.g. nuclear factor κappaB (NFκB) [18]. In addition, lipotoxicity and increased glucose concentration inhibit β-cell proliferation [17]. Thus, the pancreatic β-cell in the diabetes milieu is subject to several detrimental incidents such as OS, mitochondrial dysfunction, ER stress, and islet inflammation and epigenetic modification [18, 21, 28].

Systemic inflammatory signals as well as islet inflammation may also cause oxidative stress and activation of infiltrated macrophages [26]. It has been suggested that prolonged exposure of pancreatic islet to chronic glucolipotoxicity and ROS might trigger the intracellular production of the inflammatory cytokines specifically IL-1β and TNF-α and trigger signal transduction pathways, such as NFκB resulting in ER stress, to induce expression of proinflammatory genes, mitochondrial dysfunction, secretory dysfunction, and apoptosis. Additionally, this may also be triggered by proinflammatory signals from other organs, e.g. adipose tissue [18]. Indeed, 13 weeks of pharmacological inhibition of IL1-β in T2D patients by subcutaneous injections of the IL-1 receptor antagonist (IL-1RA) did increase β-cell secretory function [29]. This supports the hypothesis that inflammation plays an important role in the aetiology of T2D and may be causally related to β-cell dysfunction in T2D.

The aetiology, pathophysiology, and treatment of T2D are undeniably multifactorial and the understanding of T2D is increasing rapidly, but reducing obesity remains essential to improve β-cell function. However, a residue β-cell capacity appears to be essential for remission emphasising the need for lifestyle intervention early in the clinical management [20]. While exercise is less recognised as an efficient therapy for weight loss, dietary therapy is [30]. With the recent advantages in the role of very low-calorie diets on β-cell function [20, 31], it is important to study the role of exercise therapy in combination with dietary-induced weight loss to fully understand the implications for patient care. An outline of the current understanding of the effects of exercise training on the β-cell and the mechanisms leading to improved β-cell function is discussed in the following section.

### Exercise and inflammatory factors in β-cell dysfunction

It has previously been suggested that the anti-inflammatory effects of exercise may partly be linked to improved β-cell function in T2D. This may be due to mechanisms that are different from diet-induced weight loss [32]. Such an example is IL-6 secreted from contracting skeletal muscle, inducing an increase in the production of IL-1RA and IL-10, thus exerting a systemic anti-inflammatory effect. Moreover, IL-6 regulates visceral fat lipolysis [33], which potentially reduces systemic inflammation, while also contributing to non-insulin-dependent glucose uptake in skeletal muscle during acute exercise [34]. Also, IL-6 increases the incretin glucagon-like peptide-1 (GLP-1) that may protect β-cell from apoptosis, promote β-cell growth, and delay gastric emptying [35–37], thereby indirectly decreasing postprandial insulin demand. Hence, exercise-induced anti-inflammatory effects and myokine secretion could indirectly contribute to β-cell rest.

It is evident that hyperglycaemia induces overproduction and expression of advanced glycation end-products (AGEs) and the receptor for AGE (RAGE). Activation of the AGE-RAGE-axis has been associated with the development of diabetic complications [38, 39]. A common feature and diagnostic marker for T2D is postprandial hyperglycaemia, and a large portion of patients with T2D express a high degree of glycaemic variability (GV) in response to a meal. GV has been suggested to be more strongly associated with OS and vascular endothelial dysfunction than sustained chronic hyperglycaemia [8–10]. A proposed mechanism is that an acute increase in blood glucose (e.g. postprandial hyperglycaemia) activates the AGE-RAGE-axis via reactive oxygen species and signal transduction pathways (i.e. NFκB pathway), producing OS and inflammation [38]. The increased production of ROS will further enhance the production of AGEs, generating a feed-forward cycle. Endogenous soluble forms of RAGE (esRAGE and sRAGE) are found in the circulation and are suggested to modulate the AGE-RAGE response, acting as decoy ligands [38]. Soluble RAGE might increase in response to elevated AGE-RAGE levels and act as a marker for cardiovascular disease. However, it has been shown that exercise training increases sRAGE while markers of OS decrease [40]. Inhibition of the AGE-RAGE axis thus seems imperative in reducing the risk of vascular complications and might also mitigate the production of systemic OS, offering an indirect manner to decrease the inflammatory exposure on the β-cell. GV may be reduced by exercise in patient with T2D [41–43]; still, the effect of exercise on the AGE-RAGE-axis is not fully understood, nor is the role of GV in this context.

### β-cell function in response to exercise

In a recent study from Heiskanen et al., it was observed that only 14 weeks of exercise decreased pancreatic ectopic lipid accumulation and improved β-cell function in both participants with and without T2D [44].

Evidence from human, animal, and in vitro models, as shown in a recent review, supports that exercise may increase β-cell mass (i.e. β-cell proliferation, β-cell apoptosis, and β-cell viability) and improve β-cell function (i.e. glucose sensing, insulin secretion, and insulin content) [45, 46]. However, human studies investigating exercise duration, intensity, frequency, volume, and dose dependency are few, but important for understanding the link between exercise and β-cell health [45].

### Hepatic response to exercise in relation to β-cell dysfunction

Exercise may, independently of even a minimal weight loss, relieve hepatic insulin resistance [47] and decrease hepatic fat content and de novo *lipogenesis* [48–50]. In addition, exercise improves liver fatty acid metabolism and might prevent mitochondrial and hepatocellular damage [51]. Exercise is evident as a therapeutic strategy to improve fatty liver disease and, given the crosstalk between liver and pancreas, might further potentiate the benefits of a diet-induced weight loss intervention on the β-cell.

### Skeletal muscle and glucolipotoxicity in relation to exercise and β-cell dysfunction

When plasma glucose reaches the insulin receptor, it promotes the docking and fusion of glucose transporter type (GLUT) 4, containing vesicles to the plasma membrane. Peripheral insulin resistance has been suggested to be partly mediated by an inability to oxidise excess lipid delivery/storage or convert DAG to TAG [16]. It is well established that exercise improves insulin sensitivity in peripheral tissue [34, 52], which potentially induces pancreatic β-cell rest. During an acute bout of exercise training, the exercising muscle increases glucose uptake insulin-independently via multiple mechanisms [53]. Two to 72 h post exercise, there is an increase in GLUT4 translocation to the cell membrane, increasing insulin sensitivity [54]. Also, an acute bout of exercise has been shown to induce increased diacylglycerol acyl transferase 1 expression in skeletal muscle, promoting the conversion from DAG to TAG [16]. Furthermore, an increase in muscle mass appears to be beneficial, and the combination of resistance training and aerobic training has been shown to be superior to either of the two [54]. In skeletal muscle, chronic exercise increases GLUT4 concentration and enhances capillarisation, mitochondrial function, and content, all major factors for insulin sensitivity [53]. In summary, exercise-induced effects on peripheral insulin sensitivity may therefore act as a “drain” for glucose disposal, indirectly reducing gluco- and lipotoxic exposure to the β-cell.

### Muscular changes in relation to exercise and T2D

Only little attention has been given to the negative impact of T2D on skeletal muscle. T2D, like other chronic diseases coinciding with low-grade inflammation, may lead to loss of muscle mass, resulting in reduced physical capacity and strength, a condition called diabetic myopathy [55]. Loss of muscle mass and strength is strongly associated with poor mobility and physical function [56], which subsequently leads to sarcopenia, frailty, and loss of autonomy [57]. Patients with T2D have been observed to have a 2–3-fold higher risk of sarcopenia [58]. Muscle progenitor cells (here referring to satellite cells (SC)) are essential for maintenance of muscle homeostasis and regeneration [59]. SC and endothelial cells are suggested to interact in the capillarisation of skeletal muscle [60, 61]. The microvasculature is essential for the delivery of oxygen, cytokines, growth factors, waste removal, and physical capacity of the muscle. An increased capillarisation is associated with increased SC function, and muscle perfusion may be a critical factor in repair and recovery of damaged muscles [62]. It has been demonstrated in vitro that endothelial cells in a high glucose environment secrete factors that dysregulate SC growth and differentiation [63]. In addition, autophagy is imperative for muscular regenerative capacity and function [64]. Autophagy is shown to be dysregulated in T2D, affecting e.g. myogenesis and negatively affecting the regenerative capacity of muscle [65]. However, autophagy markers are increased following exercise in humans [66] and this has been shown in both acute and chronic exposure to exercise [67]. Macrophages play an essential role in regulating muscle stem cells [68], and intramuscular inflammation has been associated with T2D. Muscle expression of macrophage genes has been linked to hyperglycaemia, whereas anti-inflammatory markers have been associated with low glycaemia, exercise, and high glucose disposal rate [69]. There is a reason to speculate that an exercise training intervention combined with diet-induced weight loss may mitigate the detrimental effects of T2D on skeletal muscle, thus maintaining muscular function and further contributing to β-cell rest. The role of exercise on autophagy in human diabetic muscle cells has to our knowledge not yet been investigated.

### Investigating the dose of exercise training in addition to diet-induced weight loss

There is evidence that higher levels of physical activity are associated with a lower mortality risk in patients with T2D [70]. However, only a few studies have focused on the effects of exercise on pancreatic β-cell function in T2D and discrepancies regarding the effect exist [46, 71–74]. The discrepancies may relate to the assessment of β-cell function [75], failure to correct for the change in peripheral insulin sensitivity, concomitant pharmacological therapy, and the pre-trial insulin secretory capacity. Moreover, exercise intensity, volume, and modality may play an essential role in the reduction of HbA1c [4, 6, 76–78]. Thus, current evidence suggests that physical activity may *directly* improve β-cell mass and β-cell function [45], and may also *indirectly* improve β-cell function and mass by inducing β-cell rest via reductions in systemic inflammation and metabolic stress (i.e. gluco- and lipotoxicity). However, evidence is limited from human studies investigating the relationship of exercise volume, intensity, frequency, and dose dependency on β-cell function [45]. As a consequence, knowledge about the exercise training dose needed to reduce micro- and macrovascular complications in T2D is almost non-existing [4, 5, 7, 79–84]. As most clinical exercise interventions in T2D base their conclusions on HbA1c, the significance of exercise training in the clinical care of prevalent T2D is challenged [5, 6, 22, 23] and investigating β-cell function with different volumes of exercise in addition to a diet-induced weight loss is of clinical relevance. We propose that combining a moderate diet-induced weight loss with exercise training may dose-dependently improve pancreatic β-cell function.

### Study objectives and hypotheses

#### Objectives

##### Primary objective

To investigate the dose dependency of exercise training on pancreatic β-cell function after 16 weeks in patients with short standing T2D.

##### Secondary objectives

To investigate the dosing effect of exercise training on mechanisms mitigating pancreatic β-cell dysfunction and markers of cardiovascular complications.

#### Research hypotheses

##### Primary

The effect of exercise training on pancreatic β-cell function (assessed as late-phase disposition index) increases with increasing volumes of exercise in combination with a diet across a 16-week intervention in patients with T2D of short duration. It is expected that both moderate volume and high volumes of exercise in combination with a dietary intervention are superior to the control intervention in improving pancreatic β-cell function.

##### Secondary

Exercise training decreases low-grade inflammation in a dose-dependent manner. The intervention-induced reductions in low-grade inflammation are associated with intervention-induced reductions in glycaemic variability, ROS, and alterations in the AGE/RAGE axis. It is expected that both moderate volume and high volumes of exercise training in combination with a dietary intervention are superior to the control or dietary intervention alone in improving low-grade inflammation, ROS, glycaemic variability, and alterations in the AGE/RAGE axis.

## Methods

### Study design and setting

**Fig. 1:**
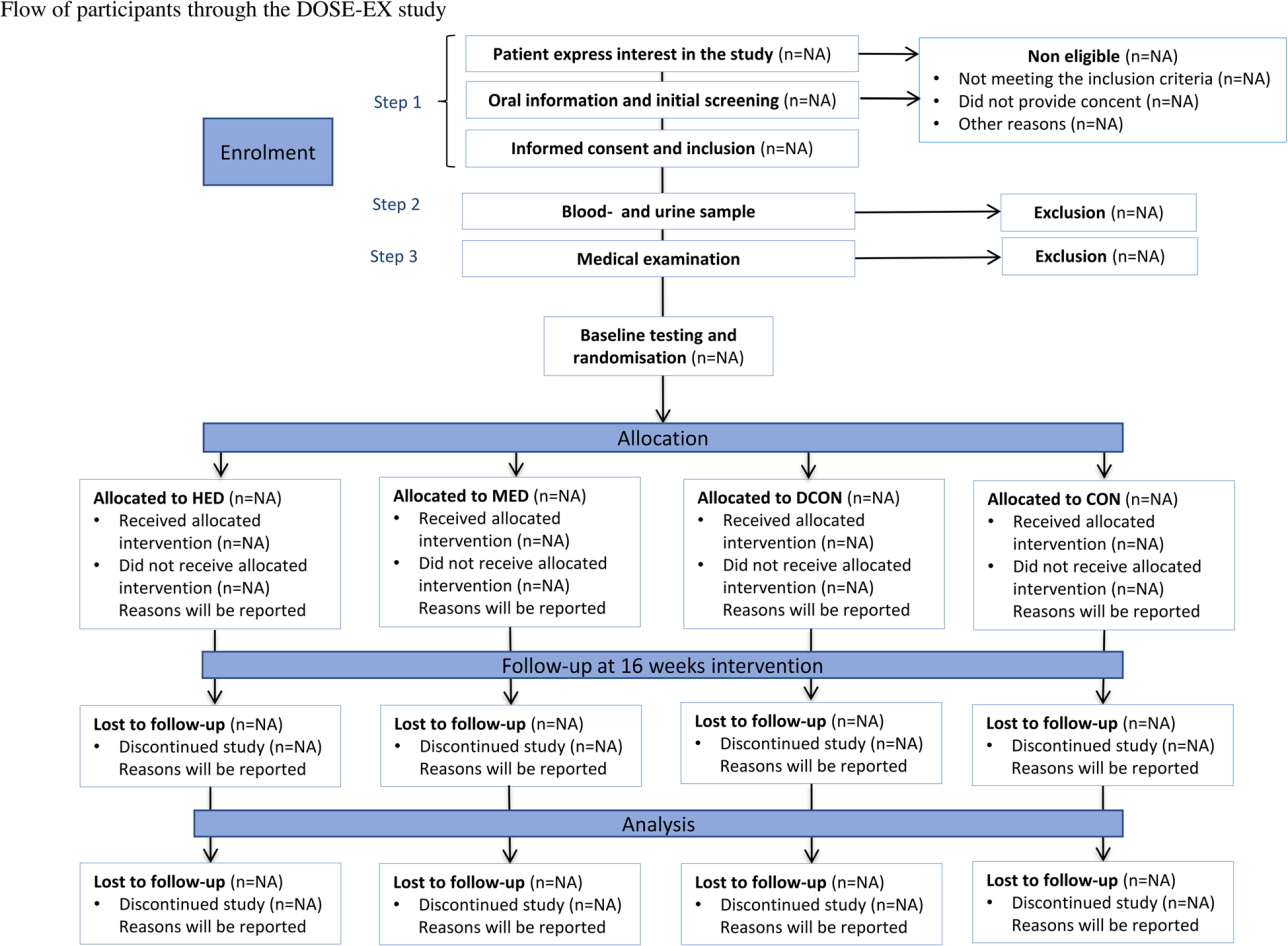
Flow of participants through the DOSE-EX study. HED, high exercise dose; MED, moderate exercise dose; DCON, dietary intervention; CON, control group. Steps 1, 2, and 3 refer to the recruitment procedure. Step 1 being the initial contact with possible participants, screening by phone and obtaining informed consent if inclusion criteria are met. In step 2, eligibility is assessed based on blood and urine samples and finally step 3 constitutes a medical examination (see text for details). If no exclusion criteria are identified the participant proceeds to baseline testing and randomisation. Participants are randomly allocated to HED, MED, DCON, or CON in a ratio of 1:1:1:1 stratified by sex

The study is designed as a parallel-group, 4-arm assessor-blinded, randomised, clinical trial where the primary outcome is β-cell function as measured by a 3-stage hyperglycaemic clamp before and after 16 weeks of intervention. Participants will be randomly allocated (1,1:1:1, stratified by sex) to four groups; (1) no intervention, (2) dietary intervention, (3) dietary intervention + moderate volume exercise, (4) dietary intervention + high volume exercise. The flow of participants is described in Fig. 1. Exercise training in combination with dietary recommendations is a cornerstone in the treatment of T2D, but the isolated effect in addition to dietary changes is poorly understood. Furthermore, knowledge is scarce on volume of exercise training needed to induce clinically significant effects. Thus, as the trial is designed to investigate the additive dose-response effects of exercise in conjunction with a dietary intervention, a multi-arm design is employed.

Participants will be recruited within the Capital Region of Denmark. The study is registered at www.clinicaltrials.gov (NCT03769883) and approved by the Scientific Ethical Committee of the Capital Region of Denmark (approval number H-18038298) prior to commencement of any study procedures. Primary place of study execution and data collection will be Centre for Physical Activity Research (CFAS), Rigshospitalet, section 7641, Tagensvej 20, DK-2200 Copenhagen (visiting address); Blegdamsvej 9, DK-2100 Copenhagen (postal address), Telephone: (+ 45) 3545 7641. Magnetic resonance imagining and magnetic resonance spectroscopy will be collected at Radiologisk Klinik X, section 3032, Rigshospitalet. All data will be collected and analysed in Denmark.

### Participants and eligibility

Poor glycaemic control and poor β-cell function prior to training predict less benefit from exercise training [85, 86]. Indeed, Dela et al. showed that T2D patients with remaining insulin secretory function improved their insulin secretory capacity with exercise, whereas participants with low secretory capacity prior to the intervention did not [46]. This is in line with observations from other lifestyle interventions in T2D [87, 88]. Moreover, to avoid any risk of drug-induced signs of hypoglycaemia or hypotension, previous trials with an expected decrease in body weight in other populations of T2D patients have adjusted the concomitant glucose- and blood pressure (BP)-lowering medications according to symptomatology and/or standard care guidelines without any adverse effects [88].

Thus, to use lifestyle as a first-line monotherapy, it is sensible to focus on T2D patients with remaining β-cell function prior to the intervention, and so, patients with a diabetes duration of < 7 years is the primary target population. Eligibility criteria are described in Table 1.

**Table 1:** Eligibility of study participants

Inclusion criteria	Exclusion criteria
Men and women aged 18–80 years	HbA1c: ≥ 75 mmol/mol with no glucose-lowering medications
Diagnosed with diabetes type 2 and/or HbA1c ≥ 48 mmol/mol if no treatment with anti-diabetic medication and/or use of anti-diabetic medication	HbA1c: ≥ 64 mmol/mol with mono glucose-lowering therapy (if compliant with the prescription)
Caucasian	HbA1c: ≥ 57 mmol/mol with ≥ dual glucose-lowering therapy (if compliant with the prescription)
No diagnose of type 1 diabetes, MODY-diabetes, type 1½ diabetes or LADA-diabetes	eGFR < 60 mL/min
T2D duration < 7 years	Macroalbuminuria at pre-screening
No treatment with insulin	Clinical or biochemical signs of hypothyroid disease
No use of sulphonylurea-based drugs	Biochemical sign of other major diseases
Body Mass Index (BMI) > 27 kg/m^2^ and < 40 kg/m^2^	Presence of circulating glutamatdecarboxylase anti body (GAD) 65
No known or signs of intermediate or severe microvascular complications to diabetes (retino-, neuro- or nephropathy)	Objective findings that contraindicates participation in intensive exercise (Pedersen and Saltin 2006)
No known cancer	Anamnestic findings that contraindicates participation in the study (Pedersen and Saltin 2006)
No lung disease, other than asthma that can be managed with beta2-agonists and does not exhibit seasonal variation.	Unable to allocate the needed time to fulfil the intervention
No known cardiovascular disease	Language barrier, mental incapacity, unwillingness or inability to understand and be able to complete the interventions
No known hyperthyroid disease	HbA1c: ≥ 75 mmol/mol with no glucose-lowering medications
No changes in hypothyroid disease treatment within the last 3 months prior to enrolment	HbA1c: ≥ 64 mmol/mol with mono glucose-lowering therapy (if compliant with the prescription)
No known liver disease—defined as ALAT or ASAT elevated three times above upper limit.	HbA1c: ≥ 57 mmol/mol with ≥ dual glucose-lowering therapy (if compliant with the prescription)
No known autoimmune disease	eGFR < 60 mL/min
No psoriasis disease requiring systemic treatment or cutaneous elements bigger than a total area of 25 cm2	Macroalbuminuria at pre-screening
No other endocrine disorder causing obesity	Clinical or biochemical signs of hypothyroid disease
No current treatment with anti-obesity medication	Biochemical sign of other major diseases
No current treatment with anti-inflammatory medication	Presence of circulating glutamatdecarboxylase anti body (GAD) 65
No weight loss of > 5 kg within the last 6 months	Objective findings that contraindicates participation in intensive exercise (Pedersen and Saltin 2006)
No changes in symptoms or anti-depressive medication 3 months prior to enrolment.	Anamnestic findings that contraindicates participation in the study (Pedersen and Saltin 2006)
No diagnose of psychiatric disorder or treatment with anti-psychotic medication	Unable to allocate the needed time to fulfil the intervention
No history of suicidal behaviour or ideations within the last 3 months **prior** enrolment	Language barrier, mental incapacity, unwillingness or inability to understand and be able to complete the interventions
No previous surgical treatment for obesity (excluding liposuction > 1 year prior to enrolment)	
Not pregnant/considering pregnancy	
No functional impairments that prevents the performance of intensive exercise	
Accept of medical regulation by the study endocrinologist	
Inactivity, defined as < 1,5 h of structured physical activity per week at moderate intensity or cycling < 30 min/5 km per day at moderate intensity (moderate intensity = out of breath but able to speak)	
No participation in other research intervention studies	

### Interventions

**Fig. 2:**
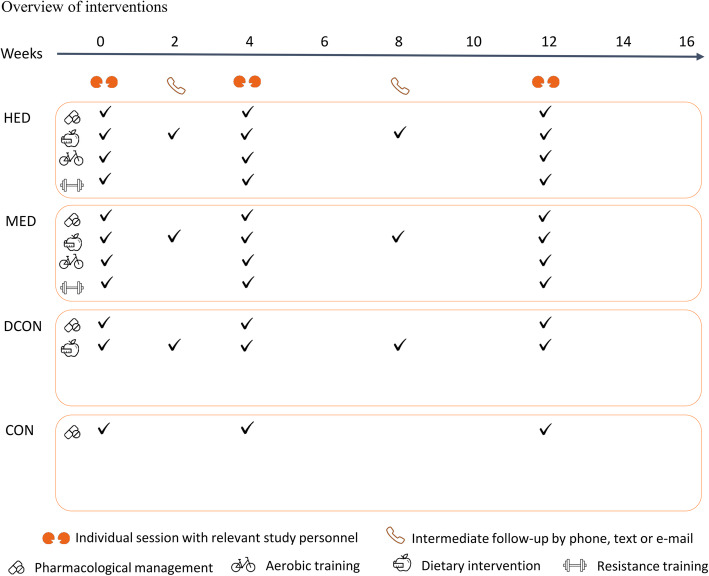
Overview of interventions. HED, high exercise dose; MED, moderate exercise dose; DCON, dietary intervention; CON, control group. The intervention runs over a full 16 weeks period. During the 16 weeks, adherence and individual adjustment in the different parts of the intervention (exercise, diet, and pharmacological management) will be evaluated by relevant study personnel. Dietary consulting is provided by the dietician. The coordinating exercise trainer handles consults regarding the exercise intervention and pharmacological management is provided by the study nurse in close collaboration with the blinded endocrinologist. Prior to the dietary consults, each participant will be asked to complete a 3-day dietary record to frame the conversation. Pharmacological adjustments will be based on resent results from blood samples and blood pressure measured at home

**Fig. 3:**
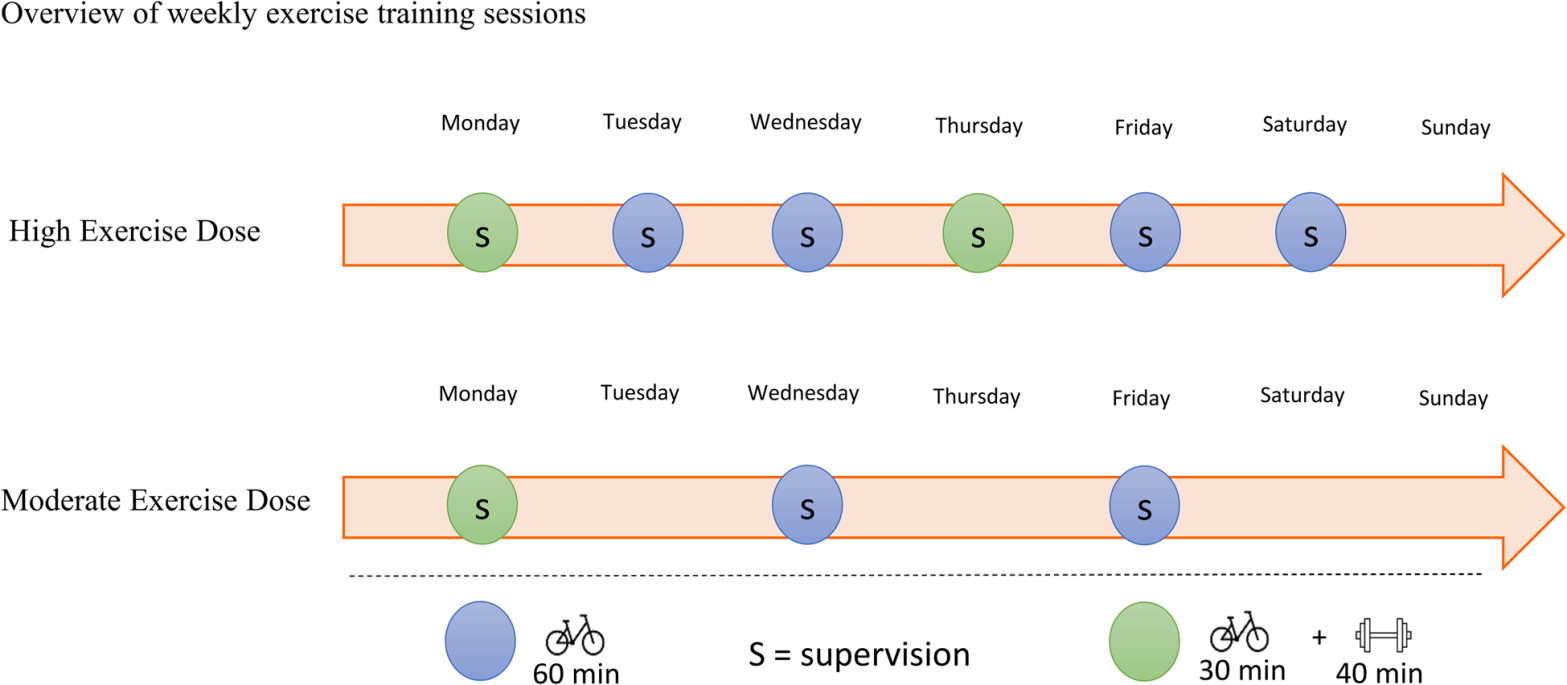
Overview of weekly exercise sessions. HED, high exercise dose; MED, moderate exercise dose; DCON, dietary intervention; CON, control group

The general intervention is based on a previous trial and adapted to the aim of this study [89, 90]. The active interventions will consist of two main components; (1) structured supervised exercise and/or (2) a dietary intervention aiming at a weight loss. Whereas there will be no difference in the dietary intervention between the lifestyle groups, the volume of physical activity and structured exercise vary according to the frequencies of the structured exercise sessions. The anticipated flow of participants is described in Fig. 1 and an overview of the intervention is depicted in Fig. 2 while a detailed description of the weekly training sessions is found in Fig. 3.

1. Control group (CON): no intervention. Participants will be encouraged to maintain habitual exercise and dietary habits throughout the study.
2. Dietary control (DCON): dietary intervention. Participants will be encouraged to maintain habitual exercise habits throughout the study.
3. Moderate exercise dose (MED): two aerobic training sessions per week of 45–60 min duration and one session per week with combined aerobic (30–35 min) and resistance (30 min) training, and a dietary intervention.
4. High exercise dose (HED): four aerobic training sessions per week of 45–60 min duration and two sessions per week with combined aerobic (30–35 min) and resistance (30 min) training, and a dietary intervention.

### Dietary intervention

Overall, the dietary intervention aims for weight loss and improved glycaemic control, while mimicking the general dietary recommendations for persons with T2D. The macro-nutrient distributions are in line with the current guidelines from the national Diabetes Association and Canadian guidelines, where individualisation in macro-nutrient distribution is within the range of 45–60E% from carbohydrate, 15–20E% from protein, and 20–35E% from fat [91]. The diet will include food items with a low glycaemic index and load, as these are associated with improved glycaemic control in contrast to food items with a high glycaemic index and high glycaemic load [92–95]. Saturated fat intake is related to cardiovascular disease risk [96]; thus, the dietary plan will aim at reducing saturated fat intake < 7E% [97] in accordance to national guidelines. Both prevention and a successful management of type 2 diabetes are highly related to diets rich in whole grains, fruit, vegetables, nuts and legumes, and lower on refined grains, red or processed meat, and sugar-sweetened beverages [96]. Therefore, a range of macro-nutrient composition will allow for individual preferences to be included in the dietary plan.

#### Dietary procedure

A registered clinical dietician will prepare the individual meal plan with proposed recipes based on individual counselling (3 sessions during the intervention). The implementation and potential adjustments will be performed continuously based on self-reported, 3-day, dietary records (for details, please refer to patient-reported outcomes) that are discussed at the individual sessions. The meal plan will cover three main meals and three snack meals per day. The content of the recipes may be adapted to individual participant preferences. Energy requirement will be based on the age-adjusted Oxford equations as described by Henry 2005 [98], aiming at a weight loss, Table 2. The participants’ body weight will be used for calculation of the energy requirement if the body mass index is < 30 kg/m^2^. If the BMI is > 30 kg/m^2^, the body weight in the equation will be adjusted to corresponding to a BMI = 25 kg/m^2^. In order to reduce the risk of mild hypoglycaemia (e.g. trembling, heart racing, feeling uncomfortable, nausea) and hunger on days with training sessions, 100–200 kcal snack meal will be added to the energy intake just before and after training (MED and HED). Furthermore, a main meal 2–3 h before a training session will be advised. In case of subjective signs of low blood glucose, the participants will be instructed to either eat one piece of fruit or to drink a glass of juice in combination with a piece of rye or crisp bread. To facilitate adherence, the dietician will contact the participants by text, phone, or e-mail (depending on their preferences) in between the individual sessions. Furthermore, participants will be allowed to contact the dietician by e-mail once a week if they have issues regarding implementation of or concerns about the meal plan.

**Table 2:** Daily energy requirement

Calculated BMR	Energy requirement per day
< 1200–1249 kcal	1200 kcal
1250–1349 kcal	1300 kcal
1350–1449 kcal	1400 kcal
1450–1549 kcal	1500 kcal
1550–1649 kcal	1600 kcal
1650–1749 kcal	1700 kcal
1750–1849 kcal	1800 kcal
1850–1949 kcal	1900 kcal
1950–2049 kcal	2000 kcal
2050–2149 kcal	2100 kcal
2150–2249 kcal	2200 kcal

Basic metabolic rate (BMR) is calculated from the age-adjusted Oxford equation as described by Henry 2005 (NNR, 2012). Participants current body weight is used if body mass index (BMI) < 30 kg/m^2^ and in case of BMI > 30, the body weight in the equation is adjusted to equal BMI = 25 kg/m^2^. Daily energy requirement is based on the level of kilocalories closest to the calculated BMR

In addition, participants in the high exercise dose (HED) and moderate exercise dose (MED) groups receive 200 kcal for restitution on days with training sessions

#### Rescue procedure for the dietary intervention

If a participant exceeds ± 30% of the prescribed energy intake as assessed by the dietary records, the procedures below will be initiated. Moreover, if the participant expresses concerns about satiety, food preferences, or food preparation techniques, the procedures, described below, will be initiated. A 1-week pause will be allowed for, e.g. vacation or illness, where the participants will receive dietary guidance that will be feasible to follow. Moreover, the participant will be asked to complete at dietary record during the pause, in order to adjust the following dietary programme to reach the pre-specified energy intake.

The procedures for the dietary intervention include;

1. An interview regarding compliance to the meal plan will be performed, and the participant will be provided with specific guidelines to practical changes in the plan by the clinical dietician. This is done to augment adherence to food items, increase satiety, or exchange some food items to match preferences.
2. If action 1 proves insufficient, the energy intake will be increased in steps of 100 kcal/day until the level of satiety is acceptable to the participant.

### Increased structured exercise

The training protocol will be adapted based on a previous study where the T2D participants were prescribed 4 weekly sessions of 60 min aerobic training alone and 2 combined aerobic and resistance training sessions (averaging 360–420 min of exercise per week) [89]. Although mean adherence was high (82% of the planned sessions were completed), variation was high and imperfect [90]. Since the variation was high in adherence in the previous study, a lower dose of exercise training may be expected in a subset of participant in the Dose-Ex study. Because of this expectation, and because of previous analyses suggesting that there may be an inverse dose-response relationship between reductions in HbA1c and aerobic training volume [4, 6], a group of moderate dose exercise training is formed (i.e. MED group). Furthermore, high intensity during both aerobic and resistance training has been shown to have a superior effect on reduction in HbA1c compared to moderate intensity [99–101]. Thus, high-intensity training is implemented in the exercise training protocol. As the effect of exercise training on glycaemic control is more closely related to the number of training sessions [6], we will reduce the number of sessions by 50% to three sessions/week in the MED group and maintain the original session frequency in the HED group (six sessions/week).

#### Exercise procedures

**Fig. 4:**
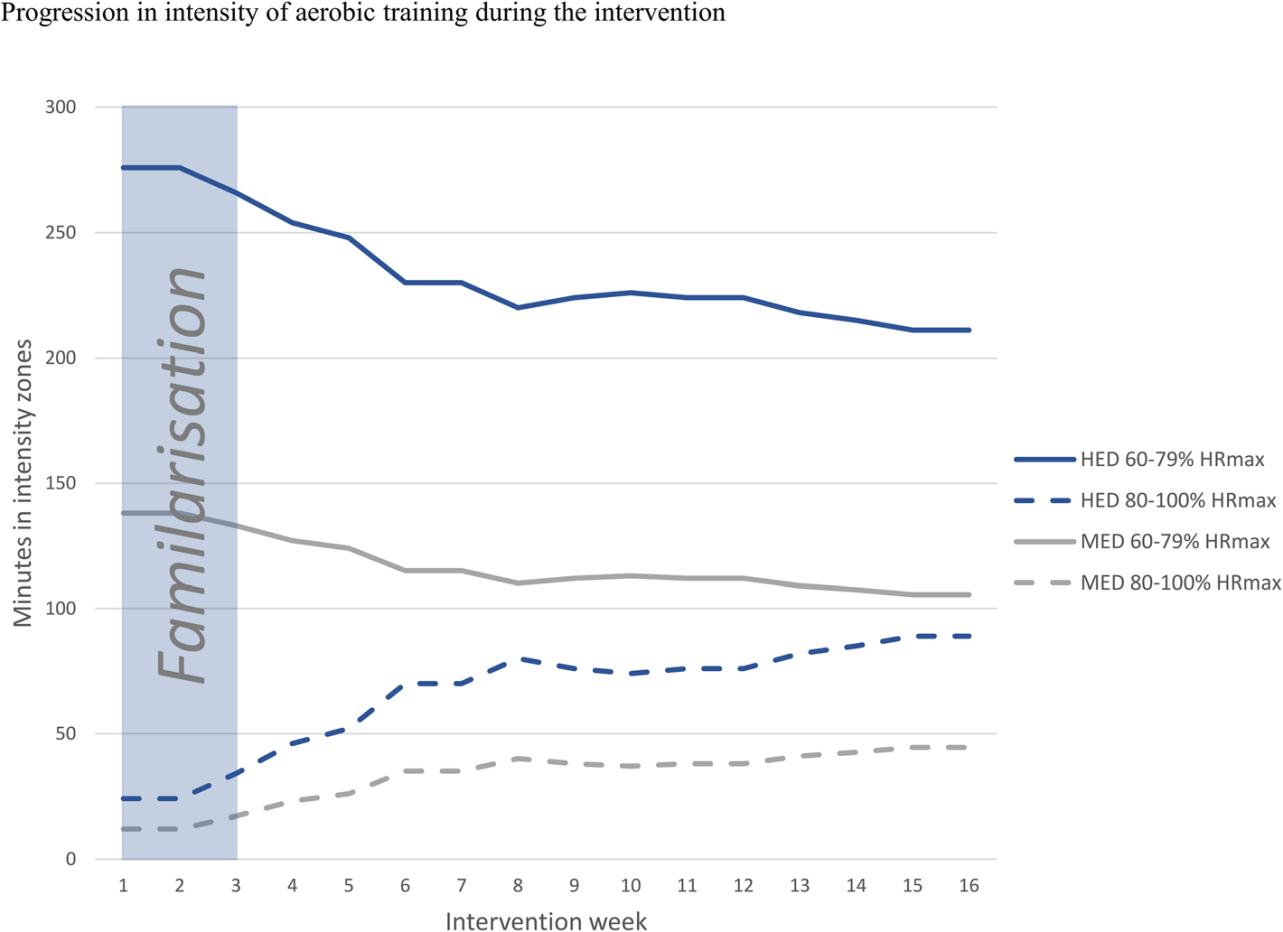
Progression in intensity of aerobic training during the intervention. During the 16 weeks, time spend in intensity zone 80–100% of maximum heart rate (HR_max_) increases gradually as time spend in intensity zone 60–79% of HR_max_ decreases. Total aerobic training volume for high exercise dose and moderate exercise dose are 300 and 150 min per intervention week, respectively. Volume remains unchanged throughout the intervention period

**Fig. 5:**
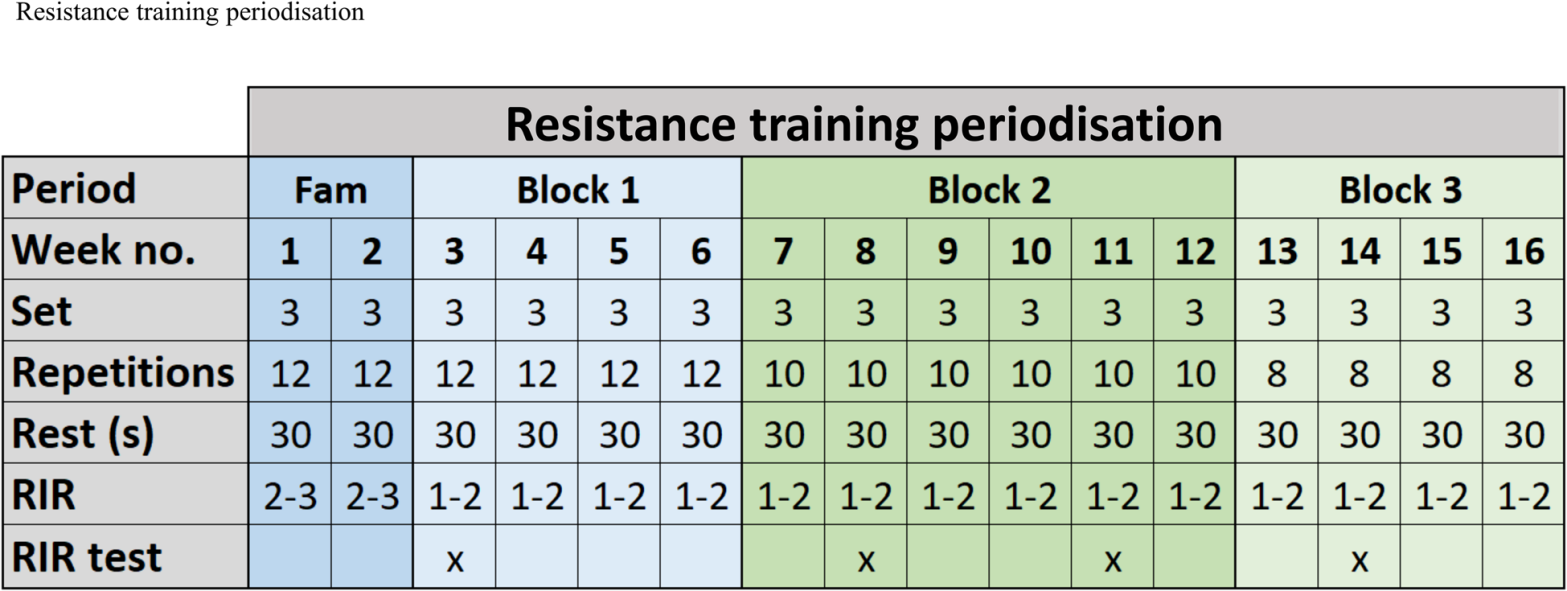
Resistance training periodisation. Fam, familiarisation period; RIR, repetitions in reserve. The initial 2 weeks of the intervention constitutes a familiarisation period with thorough instruction to each exercise and reduced intensity. The remaining intervention period is divided into blocks ensuring progression towards less repetitions with higher loads. The resistance training frequency for high exercise dose (HED) and moderate exercise dose (MED) are two and one time per week, respectively. RIR tests will be performed four times during the intervention to ensure that participants train with the prescribed intensity

In the first 2 weeks of the intervention, a familiarisation to the specific exercises will be prioritised to facilitate the training quality (i.e. to meet the prescribed training intensity) in the remaining part of the intervention. During this period, the participants will be thoroughly introduced to a heart rate monitor, training programmes, and the concept of *repetitions in reserve* (RIR) [102, 103]. The target aerobic training intensity span will be 60–100% of maximal heart rate (HR_max_), which is in line with current guidelines [2, 89, 101]. A correction of HR_max_ will be performed if a higher measurement is found during the aerobic training [104]. Throughout the intervention, the time spend exercising in intensity zone 80–100% of HR_max_ will increase and consequently the time spend exercising in intensity zone 60–79% of HR_max_ will be reduced (Fig. 4). The aerobic training programmes are found in Additional file 1 and will be programmed on the Polar HR watch (Polar V800, Polar, Holte, DK). The Polar HR watch will show the participant when it is time to modify the intensity in order to reach the target intensity zone during each training session. Replacement of the aerobic training programmes throughout the intervention will permit progression in minutes spend in intensity zone 80–100% of HR_max_. Aerobic training programme 1, 2, and 3 will be used during the initial 2 weeks. The volume and intensity of the resistance training will also be in line with current guidelines, e.g. 3–6 bouts of 8–12 repetitions per muscle group with intensities varying between 9 and 15 repetition maximum (1–3 RIR) [2]. The resistance training periodisation is presented in Fig. 5. Specific supersets with resistance training exercises can also be found in Additional file 1. If a participant can complete 3 repetitions more than prescribed (12, 10 or 8 repetitions), the load (i.e. resistance) will systematically increase in the next resistance training session (Table 3). The resistance of the exercise will be adjusted if the systematic increase in load does not fit perfectly in practise, due to the limitations regarding the different relationship between upper-/lower-body exercises and single-/multi-joint exercises [105]. To ascertain compliance to the intervention and quality of the training, all exercise sessions will be supervised by educated trainers (see below). On a weekly basis, the educated and experienced trainers will adjust the individual exercise plans to accommodate individual participant preferences in terms of exercise modality and to avoid potential overuse injuries. The aerobic training modality is individualised based on preferences, however, to minimise the risk of injuries associated with running [105], only walking, cycling, and cross-training will be allowed. In terms of resistance training, if participants report any pain or discomfort associated with a given exercise, other exercises focusing on the same muscle groups will be selected while intensity (1–3 RIR) will be assured.

**Table 3:** Progression of resistance training

RIR	Increase in load (%)	Equation
**1–2**	No change	–
**3**	7*	Current load (kg) × 1.07
**4**	10	Current load (kg) × 1.10
**5**	13	Current load (kg) × 1.13
**6**	16	Current load (kg) × 1.16
**7**	19	Current load (kg) × 1.19
**8**	22	Current load (kg) × 1.22
**9**	25	Current load (kg) × 1.25
**10**	28	Current load (kg) × 1.28

*RIR*, repetitions in reserve; *kg*, kilogrammes of weight plates on the exercise machine

The participants will take as many repetitions as possible until muscular failure in the third set during the RIR test. If the participant can complete three repetitions more than prescribed (12, 10 or 8 repetitions), the increase in load will follow the equations above and the new load will be initiated at the following resistance training session. The percentage increase in load is adapted from previous studies [102]

*Minimum increase with one weight plate corresponding to 2.5–10 kg depending on the specific exercise machine

### Modifications and strategy to maintain and improve adherence

#### Exercise intervention

Compliance will be monitored by the trainers continuously through the study. During the training sessions, the trainers will investigate whether the participants need help to handle barriers regarding the intervention initiatives. If a participant completes less than 80% of the training volume prescribed over a 1-week period, procedures to prevent drop-out will be initiated. A 1-week vacation will be allowed, where the participants will receive exercise programmes that will be feasible to complete at the vacation location. The programmes will closely mimic the assigned intervention. In case that the exercise volume is unfeasible during vacation, the volume will be reduced. Subsequently, a specific plan to reach the overall exercise volume (across 16 weeks) will be drafted in collaboration with the participant, e.g. increasing some of the existing exercise sessions by 15–30 min over a period until the target volume has been reached.

The drop-out prevention procedures for the exercise intervention groups include

1. The participant will be offered a consultation with an exercise trainer to help handling the worries and to help manage the time. If the lacking compliance relates to injuries, pain or resistance to exercise modality, the exercise modality may be altered, whereas the exercise intensity will be maintained.
2. If compliance is not corrected/maintained within a week based on action 1, a temporary adjusted plan will be made in collaboration between the trainers and participant with the aim of maintaining the weekly training volume by reducing the number of sessions of exercise per week, but increasing duration (unchanged intensity).
3. If this is not sufficient to correct/maintain compliance, the training volume will be reduced for a short period of time by retracting 1/3 of the exercise sessions per week for 2 weeks. During this process, a plan to restore the training volume will be formed in collaboration between the trainer and the participant.

#### Adherence assessment

Posture allocation and physical activity behaviour will be measured using three-axial accelerometer-based physical activity monitors (Axivity AX3, Newcastle, UK). All heart rate profiles will be recorded during the exercise interventions (Polar V800, Polar, Holte, DK). As multiple modalities will be allowed to target the muscle groups described in the training plan, all participants in the active group will receive a sheet with pictograms of possible exercises available in the training centre (see Additional file 1 for an example). The final sheets will be formed when final training locations are identified. The participant will note down the resistance and RIR after each exercise modality. Moreover, if the participant does not complete or only partially completes the session as prescribed, the proportion of completion and reasons for not full completion will be noted on the sheet. Structured and systematic RIR testing will be performed to ascertain the planned resistance training intensity (see Fig. 5). In periods between RIR testing, the trainers will randomly perform RIR tests to check the validity of the self-reported RIR during the sessions and adjust the load if needed.

#### Education of intervention personnel

Trainers who study sports science or physiotherapy will be recruited for the training intervention and a registered clinical dietician will be recruited for the dietary intervention. The primary working tasks of the trainers and the clinical dietician will be to assure compliance with the protocol and to prevent loss-to-follow-up. The trainers will also be responsible for motivating participants and making individual adjustments to the participants’ exercise training programmes in order to reduce the risk of injuries. All intervention personnel will partake in meetings, presentations, and discussions led by the principal investigators and clinicians, which will cover the following:

- The research protocol
- Disease pathophysiology (type 2 diabetes mellitus)
- The intervention: organisations and collaborators, exercise selections, physical activity, and diet
- Motivation
- Potential medical issues during the intervention (including a course in cardiopulmonary resuscitation)

### Concomitant care (all participants)

#### Pharmacological management procedures and algorithm

At visits 1, 4, and 5, biochemical markers of glucose control, lipids, and blood pressure will be assessed by the study endocrinologist, who will be blinded for subject allocation. Pharmacological management will be conducted in conjunction with self-reported symptoms of hypotension (e.g. dizziness, especially at standing up from a sitting/laying position, confusion, and fatigue) or subjected signs of hypoglycaemia (e.g. hunger, trembling, heart racing, nausea, and sweating). Pharmacological management will follow the trial algorithm for treatment targets and pharmacological titration (Additional file 2). Lipid-lowering treatment will continue at any given dosage if LDL cholesterol is < 2,5 mmol/L at inclusion. The study endocrinologist will manage the medication in accordance with the pre-defined algorithms. All changes in pharmacological treatment and adherence will be logged for later analysis. The participants will moreover be informed about side effects as well as subjective signs of increased hypo- or hyperglycaemia (thirst, fatigue, polyuria, confusion) or hypotension and urged to contact the study nurse in case of any adverse symptoms. Safety criteria will include adverse events, health-related outcomes (e.g. symptoms resembling episodes of angina pectoris or signs of cardiac arrhythmias) and participant-reported hypoglycaemic episodes (plasma glucose < 4 mmol/l, see below). Minor hypoglycaemic episodes will be defined as those that can be self-treated; major episodes will be defined as plasma glucose < 3 mmol/l or episodes requiring third-party assistance or medical intervention. In case of unacceptable adverse effects, medication will be changed according to titration described earlier. In case of participant-reported (see above) symptoms of hypo- or hyperglycaemia or hypotension, the participant will be asked to measure blood glucose using a blood glucose metre (for 3 consecutive days: morning (fasting), before evening meal, and 2 h after evening meal (postprandial)) and blood pressure profiles (18 home-based resting measurements across 3 days with 3 measurements in the morning and evening). The blood glucose and blood pressure profiles will be assessed by the nurse and presented to the study endocrinologist in a blinded manner, and the endocrinologist will manage the pharmacological treatment based on the algorithm.

### Study endpoints

An overview of outcome assessment is found in Table 4.

**Table 4:** Outcome assessment during the intervention (SPIRIT figure)

Participant timeline	Week	− 8 >	− 2	− 1	0	4	12	17	18	18
	Domain	Visit 0	Visit 1	Visit 2	Visit 3	Visit 4	Visit 5	Visit 6	Visit 7	Visit 8
**Intervention period**					X	X	X			
**Primary outcome**										
Hyperglycaemic clamp	β-cell function			X					X	
**Secondary and exploratory outcomes**										
Clinical blood sampling	Clinical, functional, metabolic markers of mechanisms^1^	X	X			X	X	X		
Urine sampling	Systemic markers of oxidative stress^2^		X			X	X	X		
Mixed meal tolerance test	Glycaemic control during mixed meal tolerance test^3^		X					X		
Continuous glucose monitoring	24-h glucose profile		X					X		
Muscle and fat biopsies	Muscle and fat profiling^4^			X					X	
*Cardiovascular procedures*										
Home blood pressure	Resting systolic and diastolic blood pressure		X			X	X	X		
*Body composition*										
Magnetic resonance imaging	Visceral fat mass				X					X
Magnetic resonance spectroscopy	Pancreatic and hepatic fat deposition				X					X
Dual-energy X-ray absorptiometry	BW, BMI, LBM, FM		X					X		
*Physical function*										
Cardiorespiratory fitness	Maximal aerobic capacity		X					X		
Muscular strength	1RM		X					X		
Physical activity behaviour	Accelerometer-based physical activity monitors		X			X	X	X		
*Patient-reported outcomes*										
Mental and physical well-being	SF36 questionnaire			X					X	
Quality of life	SF36 questionnaire			X					X	
Satiety	VAS		X					X		
Dietary records	3-day record			X		X	X	X		

*BW*, body weight; *BMI*, body mass index; *LBM*, lean body mass; *FM*, fat mass; *1RM*, one-repetition maximum; *VAS*, visual analogue scale

1. Total cholesterol, low- and high-density lipoprotein, HbA1c, interferon-ɣ, interleukin 10, interleukin 8, interleukin 6, interleukin 1, tumour necrosis factor α, Advanced Glycation End-products (AGE), receptor for AGE (RAGE).

2. 8-oxoGuo and 8-oxodG

3. iAUC, tAUC of glucose, insulin, glucagon and C-peptide, circulation markers of appetite regulation and gastric emptying.

4. Muscle progenitor cell isolation, snap freeze and tissue-tek.

#### Primary outcome

The between groups differences for change in the late-phase disposition index (DI) from baseline (0 weeks) to follow-up (16 weeks) during the final 30 min of the hyperglycaemic phase of the hyperglycaemic clamp.

#### Secondary outcomes

Secondary measurements of pancreatic β- and α-cell function, post prandial glycaemic control, visceral and organ-specific fat content, body anthropometrics, blood glucose control, blood lipids, blood pressure, gastric emptying, physical fitness, and incretin response. All outcomes designated for the primary article on β-cell function are listed in Table 5.

**Table 5:** Overview of outcomes and variables designated article 1 concerning β-cell function

Outcome	Time frame	Domain	Measurements
*Primary*	Baseline and after 16 weeks	β-cell function	Late-phase DI from hyperglycaemic phase
*Secondary*	Baseline and after 16 weeks	Secondary measures of pancreatic α- and β-cell function.	Hyperglycaemic clamp • GLP-1 stimulated insulin, C-peptide and glucagon secretion • Arginine stimulated insulin, C-peptide and glucagon secretion • 1st phase C-peptide and insulin secretion defined as the peak concentration during the initial 10 min of the hyperglycaemic clamp • Late-phase S_I_ (mean Glucose infusion rate over last 30 min of clamp phase/ (mean insulin × glucose)) • Early phase DI (DI from 0 to 30 min) • Rate of glucose disappearance (*R*_d_) • Rate of glucose appearance (*R*_a_)
*Secondary*	Baseline and after 16 weeks	Post prandial glycaemic control	Mixed meal tolerance test (MMTT) • iAUC of glucose, insulin, glucagon and C-peptide • tAUC of glucose, insulin, glucagon and C-peptide • Indices of insulin secretion, insulin sensitivity and β-cell function
*Secondary*	Baseline and after 16 weeks	Visceral and organ-specific fat content	Magnet Resonance (MR)
*Secondary*	Baseline and after 16 weeks	Body anthropometrics	• Body weight • Body mass index • Lean body mass • Total fat mass
*Secondary*	Baseline, 4 weeks, 12 weeks and after 16 weeks	Blood glucose control	• HbA1c • Fasting glucose • Fasting C-peptide and insulin
*Secondary*	Baseline, 4 weeks, 12 weeks and after 16 weeks	Blood lipids	• Total cholesterol • Triglyceride • Low- and high-density lipoprotein
*Secondary*	Baseline, 4 weeks, 12 weeks and after 16 weeks	Blood pressure	• Resting systolic and diastolic blood pressure
*Secondary*	Baseline and after 16 weeks	Gastric emptying	Mixed meal tolerance test (MMTT) • Rate of appearance of paracetamol
*Secondary*	Baseline and after 16 weeks	Physical fitness	• Maximal aerobic capacity (VO_2_ peak) • One-repetition maximum (RM) strength
Secondary	Baseline and after 16 weeks	Incretin response	Mixed meal tolerance test (MMTT) • Blood levels of incretins

*iAUC*, incremental area under the curve; *tAUC*, total area under the curve; *DI*, disposition index

#### Other outcomes

Other outcomes designated to investigate the effects on glycaemic variability, markers of systemic oxidative stress, low-grade inflammation, and markers of glycation are depicted in Additional file 3. These outcomes will be included in the article, assessing systemic oxidative stress and diabetic complications.

### Data collection methods

#### Precautions prior to testing

In order to avoid any interferences of drugs, physical activity, etc. on the various measurements, the participants will be informed about the following restrictions:

Visits 1, 2, 6, and 7

- All glucose-lowering drugs and cholesterol-lowering drugs must be discontinued for 48 h
- Fasting must be initiated at least 10 h prior to testing
- No exercise 36–48 h prior to testing
- No caffeine 24 h prior to testing
- No alcohol 48 h prior to testing
- No smoking 8 h prior to testing
- No Antacida, NSAIDs, paracetamol, or PPIs 24 h prior to testing

Visits 4 and 5

- All exercise must be discontinued 36 h prior to testing
- No caffeine 24 h prior to testing
- No alcohol 48 h prior to testing
- No smoking 8 h prior to testing

### Training and certification plans

All testing except the MRI and MRS will take place at the Centre for Physical Activity Research, Rigshospitalet Section 7641, Blegdamsvej 9, DK-2100 Copenhagen. All test personal will receive extensive training in all relevant standard operating procedures. Moreover, relevant staff will receive training and certification in DXA scanning.

### Primary outcome and key secondary outcome

#### Hyperglycaemic clamp

**Fig. 6:**
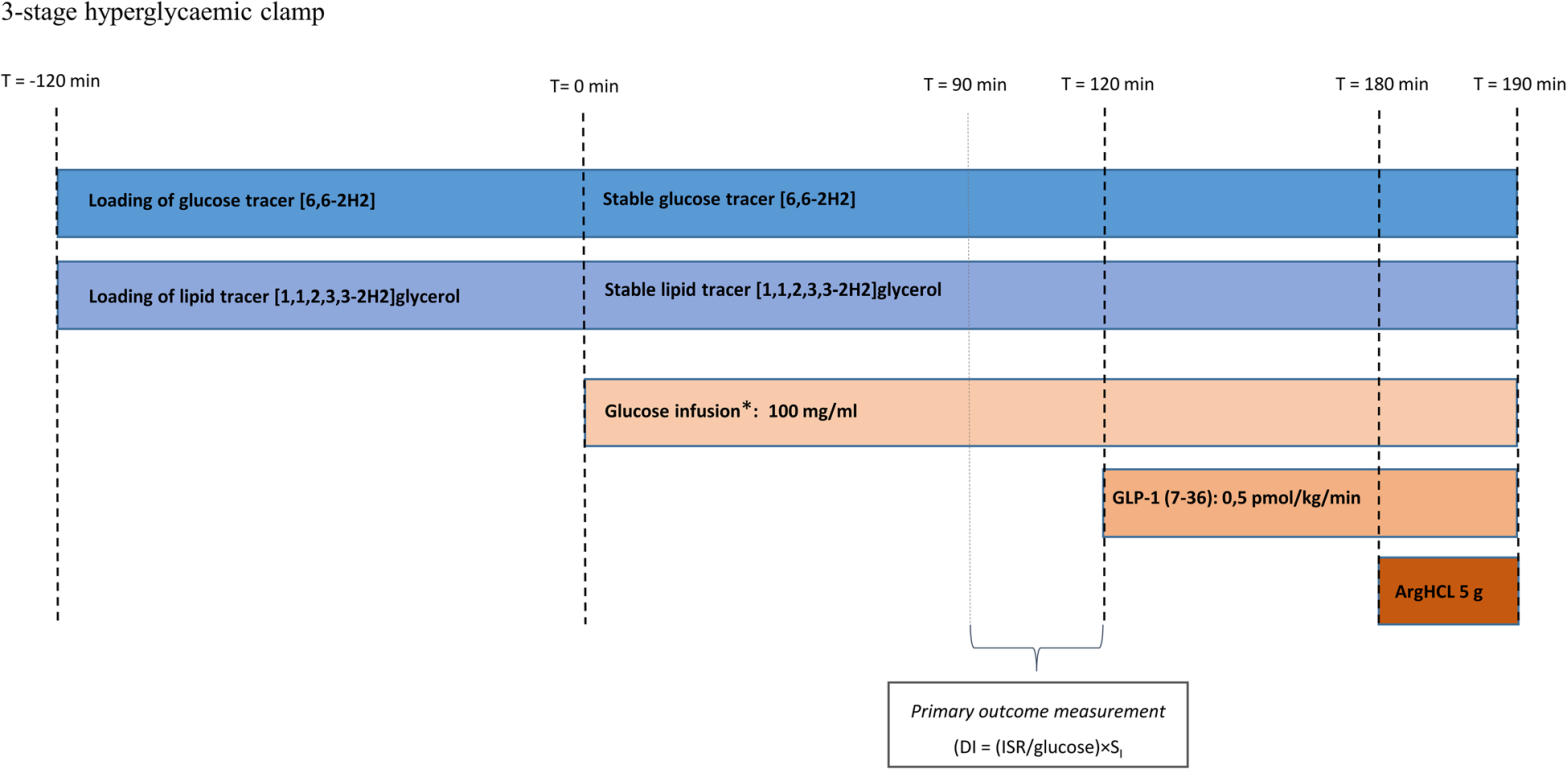
Three-stage hyperglycaemic clamp. *Glucose infusion aiming at blood glucose = 5.4 mmol/l above basal. T = time. The hyperglycaemic clamp is used to evaluate beta-cell function. Participants will meet fasting in the laboratory in the morning. After placement of an antecubital venous catheter in both arms, a tracer prime dose (concentration calculated from fasting glucose and body weight) will be injected and infusion of tracers will commence at T = − 120 min. Tracer infusion will continue for 2 h to reach saturation in the circulation. During the 2-h tracer loading period, the muscle and adipose tissue biopsies will be collected. At baseline (time 0), the glucose infusion will be initiated aiming to reach blood glucose concentration at 5.4 mM above fasting. Simultaneously, the tracer infusion rate will be adjusted accordingly. Blood glucose will be measured every 5th minute throughout the remaining clamp and glucose infusion rate (GIR) will be adjusted according to the algorithm. At T = 120 min, the GLP-1 infusion is added. After another hour (T = 180), the arginine bolus is administered, and blood samples are taken continuously for 10 min before ending the clamp

For the 3-stage hyperglycaemic clamp, an antecubital venous catheter will be placed for infusion and another line will be placed in the opposite arm for blood sampling. After baseline blood sampling (*t* = − 120 min) tracer primer will be injected and infusions will be initiated as presented in Fig. 6. Glucose and glycerol will be used as tracers in order to assess rate of appearance and disappearance of glucose, and glycerol kinetics will allow us to estimate lipolysis. Glucose clamp level will be 5.4 mM above fasting glucose (post-intervention clamp level will be equal to the pre-intervention clamp level). Increase in blood glucose will be initiated by a square-wave glucose infusion lasting 15 min. After this, glucose concentration will be kept constant and glucose infusion rates will be adjusted based on blood glucose measurements (ABL 8 series, Radiometer, Denmark), obtained every 5th min according to an automated algorithm [71]. At *t* = 120 min, the hyperglycaemic + GLP-1 stage will be commenced by infusing a primed (0.5 pmol/kg), continuous GLP-1 infusion, mimicking postprandial levels in healthy individuals. At *t* = 180 min, the arginine stage will be commenced with intravenous injection of arginine hydrochloride (5 g given over 30 s) to assess maximal insulin secretory capacity. At *t* = 190 min, the clamp will be terminated. At the start of the study day and prior to baseline sampling, participants will be asked to empty their bladder and subsequently all urine will be collected throughout the clamp. To ensure collection of the total urine volume, the participants will be asked to empty their bladder one final time just after the last blood sample (*t* = 190 min). Furthermore, spot urine samples will be collected when the participant goes to the bathroom and the time will be noted on the samples. Participants will be encouraged to keep as calm as possible during the clamp session and, if possible, refrain from bathroom breaks at times critical to outcome assessment (e.g. last 30 min of in the hyperglycaemic phase). Endogenous glucose control will be ensured before removing the catheters and allowing the participant to leave the study site. For a detailed description of blood sampling throughout the clamp, please see Additional file 4.

### Secondary and explorative outcomes

#### Clinical examination, clinical blood, and urine sampling

A medical history and examination (stethoscopy of heart and lungs, foot exam including peripheral pulse conditions, body weight, height, and electrocardiography) will be performed by standard procedures. Blood sampling will be conducted at all visits by standard procedures. The blood samples (25 ml) will be analysed for cholesterols, triglycerides, glucose, C-peptide, insulin, HbA1c, haematology, electrolytes, liver and renal status, and endocrinology (including human chorionic gonadotropin, if relevant) at the Department of Clinical Biochemistry, section 3011, Rigshospitalet. Spot urine will be collected throughout the study at the beginning of study days and frozen immediately after collection. Urine will be used to measure 8-oxo-7,8-dihydroguanosine (8-oxoGuo) and 8-oxo-7,8-dihydro-2′-deoxyguanosine (8-oxodG), using a validated method of ultra-performance liquid chromatography and tandem mass spectrometry [106].

Blood pressure will be monitored by standard procedure for home blood pressure measurements using a calibrated Microlife BP A3 Plus blood pressure monitor (Microlife AG Swiss Corporation Espenstrasse 139, CH-*9443 Widnau*/ *Switzerland).*

#### Mixed meal tolerance test

To evaluate the postprandial glucose metabolism and gastric emptying time, a standard 3-h mixed meal tolerance test (MMTT) will be performed at baseline and after the 16-week intervention (Additional file 5). After an overnight fast, serial blood samples will be drawn at baseline, 0, 15, 30, 60, 90, 120, 150, and 180 min after intake of a 420 ml liquid meal (total energy 735 kcal) consisting of 400 ml Nestlé Resource (E%: 55/30/15, carbohydrate/fat/protein respectively) with the addition of 36 g dextrose, diluted in 20 ml of water. Furthermore, 1.5 g paracetamol will be added to the MMTT for the purpose of measurement of gastric emptying time. Participants will be asked to ingest the mixed meal in less than 2 min. Blood samples will be collected in relevant tubes and analysed for, e.g. glucagon-like peptide-1 (GLP-1) (active and total), glucagon, gastric inhibitory polypeptide (GIP), insulin, pro-insulin, C-peptide, glucose, and rate of gastric emptying. The rate of gastric emptying will be calculated as previously reported [107].

#### Continuous glucose monitoring

Fourteen days of continuous glucose monitoring (CGM) will be performed using a blinded CGM sensor (FreeStyle Libre Pro, Abbot Diabetes Care Ltd., UK) inserted in the subcutaneous adipose tissue on the upper arm.

#### Biopsies

A muscle biopsy (approx. 2–300 mg) will be obtained from m. vastus lateralis using a Bergström needle. Five millilitres of lidocaine (20 mg/ml) will be administrated as local anaesthetic before the biopsy is taken. Immediately after the biopsy, skeletal muscle progenitor cells will be isolated, and the remaining muscle tissue will be divided in two. One part will immediately be frozen in liquid nitrogen and then stored at − 80 °C for protein and RNA analysis. The second muscle part will be imbedded in tissue-tek and stored at − 80 °C for histological procedures. Abdominal subcutaneous adipose tissue biopsies (up to 200 mg) will also be obtained with a Bergström needle after administrating 2 ml Lidocaine (20 mg/ml) as local anaesthetic. The adipose tissue will be divided in two. One part will be frozen in liquid nitrogen and stored at − 80 °C and imbedded in tissue-tek. The other part will be used for isolation of adipose tissue cells.

### Body composition

#### Abdominal magnetic resonance imaging (MRI) and magnetic resonance spectroscopy (MRS)

MRI and MRS will be performed using a Siemens Magnetom Prisma 3 Tesla matrix magnetic resonance scanner (Erlangen, Germany) at 3-mm intervals. All adipose tissue located from the diaphragm to the pelvic floor inside the peritoneum will be traced manually as the visceral fat region of interest. MRS to assess liver and pancreatic fat will be performed based on the MRI and analysed as described elsewhere [108]. All MR scans will be analysed by investigators blinded for subject allocation. This will be achieved via the investigators not being unblinded until the end of analysis.

#### Dual X-ray absorptiometry (DXA)

A DXA scan (Prodigy Advance, GE Medical Systems – Lunar, Madison, WI, USA) will be used to assess body fat and lean mass before and after the intervention. Participants will be in a fasting state and asked to empty their bladder prior to the scan.

### Physical function

#### Maximal aerobic capacity

The participants will undergo a maximal graded exercise test on a bicycle ergometer for evaluation of their peak oxygen consumption (VO_2peak_, ml oxygen per min per kg). The test will start with a 5-min warm up at 60 or 80 W for women and men, respectively. Warm up will be immediately followed by a 20 W increase every minute until volitional exhaustion. Subjects will be given strong verbal encouragement during the test. Oxygen consumption will be assessed using continuous indirect calorimetric measurements (CPET, Cosmed, Italy). The highest 20 s breath by breath average will be taken as the VO_2peak_ provided that standard criteria are fulfilled [109]. Maximal heart rate (HR_max_, beats per min) will be determined using a Garmin Premium heart rate monitor.

#### Muscle strength

Maximum muscle strength will be assessed in two functional exercises performed in resistance training machines (chest press, leg extension) by 1-repetition maximum (1-RM). Four warm up sets including 10, 6, 3, and 1 repetitions with very light, light, moderate, and heavy load, respectively, will be performed. 1-RM attempts will then take place. If a lift is successful, the participant will rest for 3 min before attempting the next lift with a heavier load. The investigator will use the participant’s feedback from the RIR-scale along with average velocity of each attempt to determine the subsequent attempt [110]. The load of a successful repetition will be recorded as 1-RM if an attempt with a load increase of 2.5 kg fails [103]. A failed attempt will be defined as a participant being unable to complete a lift using the proper technique through the full range of motion.

#### Physical activity

To evaluate leisure time physical activity all participants will be equipped with two accelerometers (AX3, Axivity, Newcastle, UK) for 4–6 consecutive days. One accelerometer will be placed on the right thigh, and the other will be placed on the right side of the lower back. Both accelerometers will be attached to the participant with a patch (Fixomull stretch, BSN medical, Germany).

### Patient-reported outcomes

#### Mental and physical well-being from the Short Form 36 (SF-36)

The SF-36 is a short form health survey with 36 questions. It yields an 8-scale profile of functional health and well-being scores as well as psychometrically based physical and mental health summary measures and a preference-based health utility index [111]. Moreover, socio-demographic information about education, age, ethnicity, civil status, occupation, smoking status, recreational drugs, current use or prior use of anabolic androgenic steroids, and alcohol consumption will be collected.

#### Diet record

A self-reported 3-day record of the participants’ total dietary intake will be obtained at baseline, during the intervention period (at weeks 0, 4, and 12), and after the intervention. Digital kitchen scales (Day Kitchen Scale Digital, Schou Company A/S, Nordager 31, DK-6000 Kolding) are handed out at visit 1 and the participants are asked to weigh and note down every food item in the 3-day record. Individual adjustments to the dietary intervention will be assessed according to the trial’s guidelines.

#### Satiety

Satiety will be recorded during the MMTT (immediately before, after 60 min, after 120 min, and immediately after the test) using a 5-item visual analogue scale (VAS).

### Sample size considerations

Based on a previous study with an aerobic training volume similar to the guidelines (i.e. MED group) in a population with short T2D duration, it is expected that an exercise intervention will increase late-phase disposition index derived from a hyperglycaemic clamp by 1.5 (au.) more than the control group, with a standard deviation of 1.5 (au.) of the change in the exercise and 1.0 (au.) in the control group [71]. For a contrast in a one-way ANOVA with four means (1.5, 1.0, 0.5, 0.0) and contrast coefficients (1, 0, 0, − 1) using a two-sided significance level of 0.05, assuming an error standard deviation of 1.5 and a balanced design, a total sample size of 80 participants corresponds to an approximate statistical power of 87.7%. Thus, up to 20 participants will be recruited per group (*N* = 80 in total).

### Randomisation, sequence generation, and allocation concealment

Participants will be randomly allocated, following successful completion of the baseline measurements. An independent statistician generates a computer-generated randomisation schedule in a ratio of 1:1:1:1, stratified by sex. In order to ascertain concealment, the (permuted) block sizes will not be disclosed. The schedule will be forwarded to a secretary not involved in any study procedures and stored on a password-protected computer. Sequentially numbered (according to the sequence) opaque, sealed envelopes will be prepared and stored in a locked cabinet. The envelopes will be lined with aluminium foil to render the envelope impermeable to intense light. Following the conclusion of visit 2 (V2), i.e. after the termination of the hyperglycaemic clamp, the appropriate envelope will be opened by a study nurse and the participant will be told the allocation stated on the card inside the envelope.

### Blinding

Participants will be blinded for treatment allocation until group assignment at the end of the tests on V2. However, following the baseline assessment, blinding of the participants will no longer be possible. All study personal responsible for data collection will be blinded throughout the study. The participant will be informed about group allocation by the study nurse in a closed room. The study endocrinologist managing pharmacological treatment and safety will be blinded to allocation. The clinical results will be presented to the endocrinologist by the study nurse without disclosing subject allocation. As all necessary information about intervention, medical history, and adverse events can be provided to the endocrinologist by the study nurse, the blinding of the study endocrinologist will only be repealed if considered necessary, e.g. based on symptoms of severe hypoglycaemia in relation to training or serious adverse events.

### Statistical analysis methods

#### Analysis of the primary outcome

The primary analysis will be based on the family of the intention-to-treat population, defined as the *as-observed population* (missing data will not be imputed in the primary analysis) [112, 113], and the set of participants who are as close as possible to the intended intervention protocol, i.e. per-protocol. The “Full Analysis Set” for the intention-to-treat will thus be derived from the set of all randomised participants by minimal and justified elimination of participants. Therefore, all participants allocated to an active treatment group (DCON, MED or HED) will be followed up, assessed, and analysed as members of that group irrespective of their compliance to the planned course of treatment. Sensitivity analyses will be performed using the potentially biased but conservative non-responder imputation (*baseline observation carried forward* technique) as well as the current best practice multiple imputation procedure [112]*.* Patterns of missing data will be investigated. A priori, the less restrictive missing at random (MAR) assumption is considered more reasonable than data missing completely at random (MCAR). Assuming that the data on potential dropouts are MAR, multiple imputation procedures will be applicable to handle missing data for all participants with baseline measurements.

The analyses of the primary outcome will be performed using a repeated measures analysis of covariance applied using mixed linear modelling [113, 114]. Mean change score of DI will be applied as the dependent outcome variable, whereas group (4 levels), time (2 levels), the interaction between time and group, sex (2 levels), and the baseline value of DI are included as independent variables and participant identifier as random effect. The potentially biased *per-protocol* population analysis will be adjusted for putative confounders: XXX, YYY, and ZZZ. The assumptions for using the linear models will be checked to confirm normal distribution of the residuals and the homogeneity of the variance (standardised residuals vs. the predicted values).

If the global test indicates between-group differences (*H*_0,DCON_ = *H*_0,MED_ = *H*_0,HED_ = *H*_0,CON_; *p* < 0.1), pairwise between-group differences will be explored. To maintain the family-wise type 1 error rate, a hierarchical analytic approach is engaged [115]; if we fail to progress from any of the subsequent steps (*p* > 0.05), we will interpret *p* values and CIs numerically as indicators of associations. Between-group comparisons for effect size estimation (difference in change from 0 to 16 weeks, based on a superiority assumption) will be completed in the following order:

1. CON vs. HED. If a difference is present (*p* < 0.05, 2-sided), then the next between-group comparison is performed. If not, then sequence is terminated.
2. CON vs. MED. If a difference is present (*p* < 0.05, 2-sided), then the next between-group comparison is performed. If not, then sequence is terminated.
3. CON vs. DCON. If a difference is present (*p* < 0.05, 2-sided), then the next between-group comparison is performed. If not, then sequence is terminated.
4. DCON vs. HED. If a difference is present (*p* < 0.05, 2-sided), then the next between-group comparison is performed. If not, then sequence is terminated.
5. DCON vs. MED. If a difference is present (*p* < 0.05, 2-sided), then the next between-group comparison is performed. If not, then sequence is terminated.
6. MED vs. HED.

The per-protocol population will be defined as participants (all criteria present):

1. CON
  The primary outcome is assessed at both baseline and after 16 weeks follow-up (i.e. complete case).

2. DCON
  The primary outcome is assessed at both baseline and after 16 weeks follow-up (i.e. complete case).
  Do not exceed ± 30% of the prescribed energy intake as assessed by their dietary records (assessed as the mean energy intake across that latter 16 weeks, excluding 1-week vacation administered following week 2 of the intervention)

3. MED and HED
  The primary outcome is assessed at both baseline and after 16 weeks follow-up (i.e. complete case).
  ≥ 70% of the prescribed exercise volume across the intervention period (excluding weeks 1, 2 + 1-week vacation administered following week 2 of the intervention).
  Do not exceed ± 30% of the prescribed energy intake as assessed by their dietary records (assessed as the mean energy intake across that latter 16 weeks, excluding 1-week vacation administered following week 2 of the intervention)

### Analyses of the secondary outcomes

Other continuous secondary outcomes, assessed before and after the intervention period, will be analysed by analysis of covariance (ANCOVA) with the mean change score of the variable as dependent variable and group (4 levels), sex (2 levels), and the baseline value of the variable as independent variables. Continuous variables, additionally assessed during the intervention period, will be analysed within the framework of repeated measures linear mixed models. The model includes treatment (4 levels), time (2 levels), sex (2 levels), and the possible interaction between treatment (group) and time (weeks) as fixed effects, with the baseline value of the relevant variable as a covariate and participant ID as random effect. The assumptions will be investigated as described above. Variables not meeting the model assumptions will be transformed using appropriate transformations. If no suitable transformation is identified, the median change with interquartile ranges will be reported and testing will be performed using suitable non-parametric statistical tests (e.g. Wilcoxon signed rank tests). Binary outcomes will be reported as numbers and proportions and compared using a *Χ*^2^ test or Fisher’s exact statistics.

### Retention

All participants will receive up to DKK 6000 (€800) to cover lost earnings, transport, and discomfort. The transaction will be completed upon completion of the study (all four full laboratory days (V1, V2, V6, and V7) or upon withdrawal). For every completed full day of laboratory testing, participants will receive 1.000 DKK. Moreover, DKK 500 in compensation will be added per biopsy (up to 4 in total). To prevent loss-to-follow-up amongst participants in the CON, we will offer three supervised training sessions and free membership in a fitness centre for 16 weeks following final testing.

### Data management

The web-based Clinical Trial Management System EasyTrial will be used for data entry and management (EasyTrial ApS). EasyTrial has been approved by the Danish Data Protection Board. Electronic case report forms (eCRF) and questionnaires will be generated by the sponsor in EasyTrial. Fields have been programmed with acceptable ranges for data entry. All paper material (CRF, blood screen results, questionnaires, and dietary records) will be collected and stored in a locked cabinet at CFAS, Rigshospitalet Denmark. All information from the paper material will be entered twice by in non-consecutive order into the electronic back-end system. In case of discrepancies between the entries, the original paper record will be consulted. Upon completion of the study, all paper material will be scanned and stored on the secured hospital server in an electronic form. All paper material, except for the consent form, will subsequently be destroyed. Data management will be performed using appropriate statistical software.

To enable pseudonymised data, all participants will be ascribed a unique participant identification (ID) number. The identification key (ID number to personal information) will be stored on a password-protected computer, separate from the unique ID number and the database. Printed data will be kept in a separate locked area with limited access. All patient-related information obtained during the study will be handled in accordance with the Danish law for protection of personal data (“lov om behandling af personoplysninger”) and the Danish health law (“sundhedsloven”). The blood samples will be registered from the hospital blood sample portal (Labka) and para-clinical observations will be obtained through “Sundhedsportalen”. The study has been reported to the Danish Data Protection Agency (“Region H’s paraplyanmeldelse”) VD-2018-516/ I-suite no. 6768.

### Harms, risks, and discomforts

#### Adverse events (AE) and safety evaluation

In this study, we have adopted the ICH definition of adverse event (AE) (E2A).

An AE is thus defined as; “An adverse event (AE) can therefore be any unfavourable and unintended sign (including an abnormal laboratory finding, for example), symptom, or disease temporally associated with the use of a medicinal product, whether or not considered related to the medicinal product” [116]*.*

Serious AE (SAE) is defined as; “[…] any untoward medical occurrence that at any dose: * results in death, * is life-threatening, NOTE: The term “life-threatening” in the definition of “serious” refers to an event in which the patient was at risk of death at the time of the event; it does not refer to an event which hypothetically might have caused death if it were more severe. * requires inpatient hospitalisation or prolongation of existing hospitalisation, * results in persistent or significant disability/incapacity, or * is a congenital anomaly/birth defect” [116].

AEs/SAEs (anticipated and unanticipated) will be recorded on adverse event forms. These forms will include a description and classification of the event, date of onset, date resolved, whether the event was serious or not (ICH criteria), relationship of the event to the study (1 = none, 2 = unlikely, 3 = possible, 4 = probable, 5 = definitely), action taken, and whether the study was suspended or not. All SAEs will be reported to the Regional Ethical Committee. AEs observed by any investigator and/or reported by the participant will be reported in the source data and case report form from the first (signature of informed consent) to the last protocol-specific procedure allocation [117].

##### VO_2_-max test and 1RM

Physical fitness and strength tests, where subjects must put in maximal effort. The tests can cause some degree of breathlessness and exhaustion, but both are standard methods used for scientific purposes at the CFAS laboratory.

##### DXA scan

Is not expected to cause any discomfort and involves very little radiation (0.0004 mSv), corresponding to approximately 1/10 of the radiation acquired for a thoracic X-ray. The dose is smaller than the radiation received when flying in a commercial jet from (11–12 h) SST.dk—Strålingsguiden.

##### Hyperglycaemic clamp

The hyperglycaemic clamp combined with GLP-1 infusion and arginine bolus may cause hypoglycaemic symptoms (dizziness, headache, and fatigue), following the trial. However, blood glucose will be monitored for up to 1 h after the test and a meal will be provided when testing has finished. Furthermore, there is a minor risk of infection or haematoma due to blood lines being placed. All researchers are experts in these procedures, so the risks are minimal. The hyperglycaemic clamp with GLP-1 and arginine has previously been used for scientific purposes in our laboratory. The introduction of arginine may give the participants a transient metallic taste that is short and fully reversible.

Stable isotope tracers will be used to obtain knowledge about glucose and lipid distribution. The isotopes are not radioactive and are considered safe to use.

Hyperglycaemia can lead to low blood levels of potassium. Supplementary potassium will be administered if potassium levels are low at the beginning of the clamp.

##### Blood sampling

A small peripheral venous catheter (PVC) will be placed and may cause slight discomfort and involves a small risk of local infection and oedema. The blood volume collected (maximum 965 ml/4 months) is considered too small to cause any symptoms.

##### Biopsies

The local anaesthesia can be associated with short-lasting discomfort and subjects might experience some degree of muscle pain after the biopsy. Paracetamol (1000 mg) max. Four times a day will be recommended for pain management. Generally, the fat biopsy causes much less discomfort. The procedures can leave small bruises, but normally they heal nicely. Temporary, decreased sensation at the incision area or where the local anaesthetic has been injected can be seen and heals within months. Infection occurs in 1 out of 25,000 times and may in some cases require treatment with antibiotics. Participants will be informed to contact a project physician in case of any signs of infection (heat, redness, swelling, or fever).

##### MRI/MRS

The measurements are pain free and based on radio waves and, thus, the participant is not exposed to X-rays or other sources of radiation. The scans will be performed in a tight cylinder that may cause claustrophobia. As the scans are not performed with a specific clinical purpose but rather for quantification of site-specific ectopic adipose tissue, they cannot and will not be used for diagnostic purposes. However, all scans will be screened by a trained radiologist at least 4 weeks after each scan at which occasion unexpected abnormalities may be detected. If deemed necessary and the participant so wishes (according to the consent form), the Department of Radiology, section 3024, Rigshospitalet will perform further warranted diagnostics.

##### Continuous glucose monitoring (CGM)

The method is safe and routinely used by patients with diabetes to continuously monitor the blood glucose level. Penetration of the skin involves a small risk of infection and the study participants will be informed and instructed to act in case of symptoms of infection.

## Discussion

The study is expected to result in minimal discomfort and risk for the study participants. The study examines the effects of various volumes of exercise training on pancreatic β-cell function and glucose levels in participants with short standing T2D, i.e. < 7 years. Both a rapid weight loss through diet and diet/exercise training may induce subjective signs of hypoglycaemia or hypotensive episodes. Procedures to manage medications in both the Look AHEAD and the DIRECT studies are described [87, 88]. It is thus reasonable to manage glucose- and blood pressure-lowering agents with a safety mechanism in a blinded standardised algorithm in all groups. Based on a previous study, a large proportion of the exercise training/diet groups are expected to discontinue their glucose and blood pressure-lowering medications within the study period without any adverse events [118]. Immediately following the study, the clinical parameters will be reviewed by the research physicians, and the participants will be asked to contact their general practitioner with the purpose of continuing their treatment based on the clinical guidelines [119]. Also following each participant’s final study visit, the project endocrinologist will write a summary of the participant’s clinical values and intervention and send this information to his/her general practitioner in a data-secure manner. The participants within the DCON/MED/HED groups will benefit from the study in terms of a thorough medical examination, increased physical capacity and increased T2D management. Based on previous research from our group, it is expected that a large proportion will maintain or even improve T2D control with this intervention [90]. Moreover, in contrast to the previous study, all exercise sessions are fully supervised; thus, it is expected that compliance to the lifestyle intervention will be even higher than previously reported (82%) [90]. The control group will also benefit from an extensive health check-up and achieve insight to basic anti-diabetic lifestyle alterations. After the project has finished, all participants will be re-referred to the various activities for patients with T2D in their local municipalities. Furthermore, participants in the CON group will be offered 16 weeks of supervised training after the intervention. If participants in the CON group do not wish or are unable to attend the rehabilitation programme, then a dietary plan and an extensive individualised training programme (based on the intervention provided in the DCON/MED/HED groups) will be provided.

We consider this a valuable and sound study that will contribute to the essential knowledge of possible T2D remission induced by non-surgical and non-pharmacological lifestyle intervention. Specifically, it will improve our current knowledge about if and how exercise training intervention, when administered in concert with diet-induced weight loss, affects pancreatic β-cell function in patients with T2D and possibly provides a sound alternative to conventional high-risk procedures. Moreover, the study will elucidate the time dependency and causality between the pathophysiological processes of cardiovascular damage and add to the body of evidence about how and if exercise training intervention may decrease the risk of the micro- and macrovascular complications induced by T2D. The vast amount of data collected leaves room for exploratory outcomes and, thus, hypotheses-generating studies on e.g. mitochondrial function and density, metabolomics and proteomics from urine and blood samples, circulating biomarkers of organ and/or arterial function.

In the end, the project will be an important steppingstone in the process of developing efficient lifestyle interventions with both curative and secondary prevention purposes in the clinical care of T2D.

### Trial status

The enrolment period began on 15-12-2019 and is open until *N* = 80 T2D or until 01-12-2021, whichever is reached first. However, given the present COVID19 pandemic, the time period may be extended further in order to gather *N* = 80 as the inclusion of new participants was suspended on March 13th, 2020, until May 11th, 2020 (see Additional file 6). This is protocol version number 4.

## Supplementary Information

**Additional file 1.** Aerobic training programmes and resistance training exercises completed during the intervention period.

**Additional file 2.** Algorithm for pharmacological management.

**Additional file 3.** Outcome Table Article 2.

**Additional file 4.** Hyperglycaemic Clamp Blood Sampling Schedule.

**Additional file 5.** Mixed Meal Tolerance Test.

**Additional file 6.** Suspension Note.

**Additional file 7.** Informed Consent.

## Data Availability

The data from the DOSE-EX study will be published in international peer-reviewed journals. All results will be reported according to the CONSORT guidelines [120]. Positive, negative, and inconclusive data will all be disseminated and published. All authors must comply with the “Uniform Requirements for Manuscripts Submitted to Biomedical Journals” [121]. All data are the property of CFAS and access to data will be overseen by the DOSE-EX steering committee. All members of the DOSE-EX study group will have access to the anonymised, cleaned data set upon completion of the final post-intervention testing after approval from the steering committee (based on an approved proposal or with specific reason provided). Following publication of the primary outcome, other researchers may request access to the data following an approved (by the steering committee) research proposal.

## Abbreviations

AE: Adverse event
AGEs: Advanced glycation end-products
ATP: Adenosine triphosphate
CGM: Continuous glucose monitoring
CON: Control group
DAG: Diacylglycerol
DCON: Dietary control group
DXA: Dual X-ray absorptiometry
esRAGE: Endogenous soluble receptor for advanced glycation end-products
ER: Endoplasmic reticulum
GLP1: Glucagon-like peptide-1
GLUT: Glucose transporter type
GV: Glycaemic variability
HbA1c: Glycated haemoglobin
HED: High exercise dose group
IL: Interleukin
MAR: Missing at random
MCAR: Missing completely at random
MED: Moderate exercise dose group
MMTT: Mixed meal tolerance test
MRI: Magnetic resonance imaging
MRS: Magnetic resonance spectroscopy
OS: Oxidative stress
PKCε: Protein kinase C epsilon type
PVC: Peripheral venous catheter
RAGE: Receptor for advanced glycation end-products
ROS: Reactive oxygen species
SAE: Serious adverse event
SC: Satellite cell
sRAGE: Soluble receptor for advanced glycation end-products
TAG: Triacylglycerol
T2D: Type 2 diabetes mellitus
