# Supplementary material for: The effects of different doses of exercise on pancreatic β-cell function in patients with newly diagnosed type 2 diabetes: study protocol for and rationale behind the “DOSE-EX” multi-arm parallel-group randomised clinical trial
Source: Trials. 2021 Apr 1;22:244. doi: 10.1186/s13063-021-05207-7 (PMC8017660; doi:10.1186/s13063-021-05207-7)
Supplement: Supplementary file 1 — Additional file 1. Aerobic training programmes and resistance training exercises completed during the intervention period. [file 13063_2021_5207_MOESM1_ESM.pptx]

## Slide 1
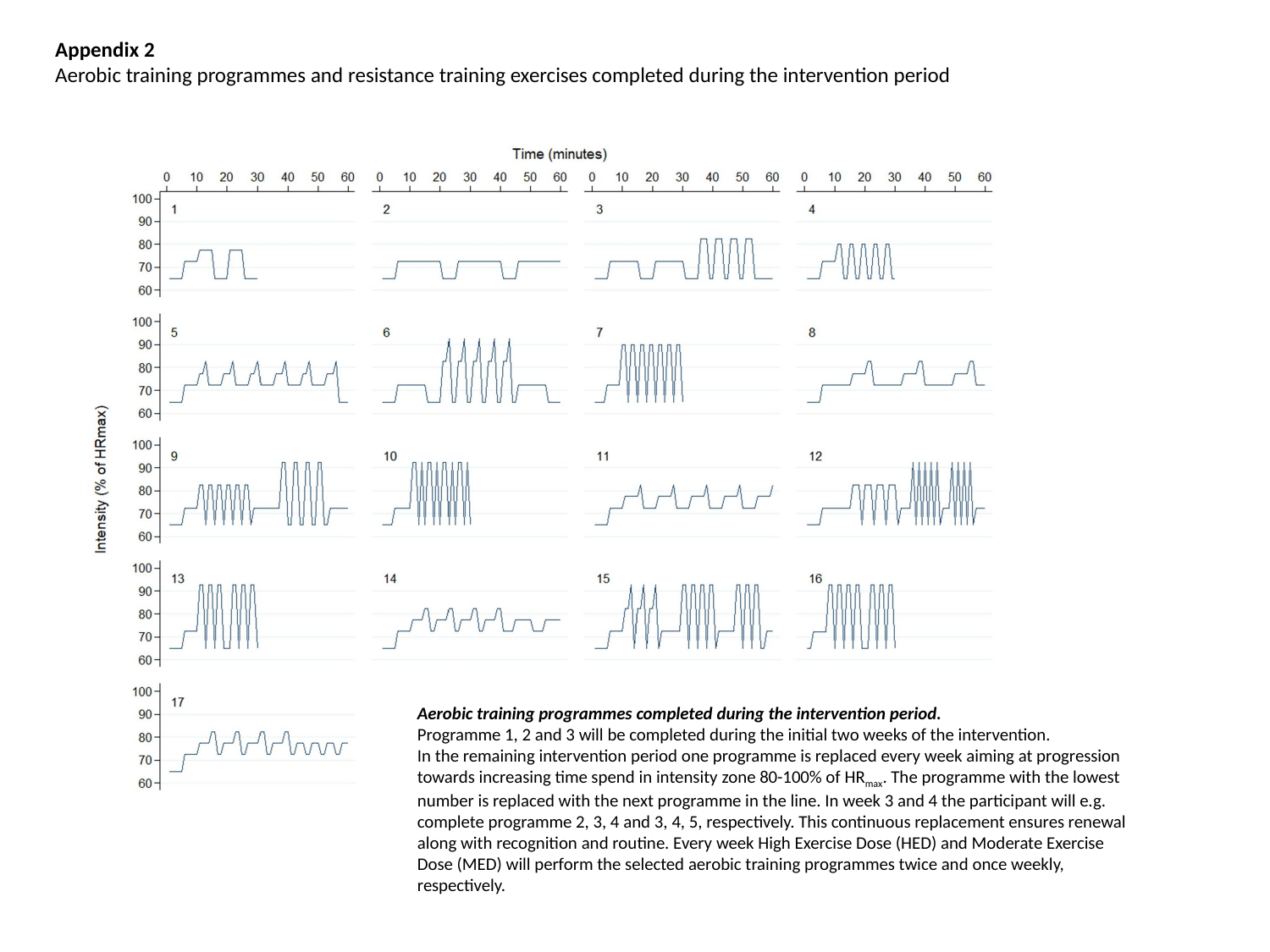

Appendix 2
Aerobic training programmes and resistance training exercises completed during the intervention period
Aerobic training programmes completed during the intervention period.
Programme 1, 2 and 3 will be completed during the initial two weeks of the intervention.
In the remaining intervention period one programme is replaced every week aiming at progression towards increasing time spend in intensity zone 80-100% of HRmax. The programme with the lowest number is replaced with the next programme in the line. In week 3 and 4 the participant will e.g. complete programme 2, 3, 4 and 3, 4, 5, respectively. This continuous replacement ensures renewal along with recognition and routine. Every week High Exercise Dose (HED) and Moderate Exercise Dose (MED) will perform the selected aerobic training programmes twice and once weekly, respectively.

## Slide 2
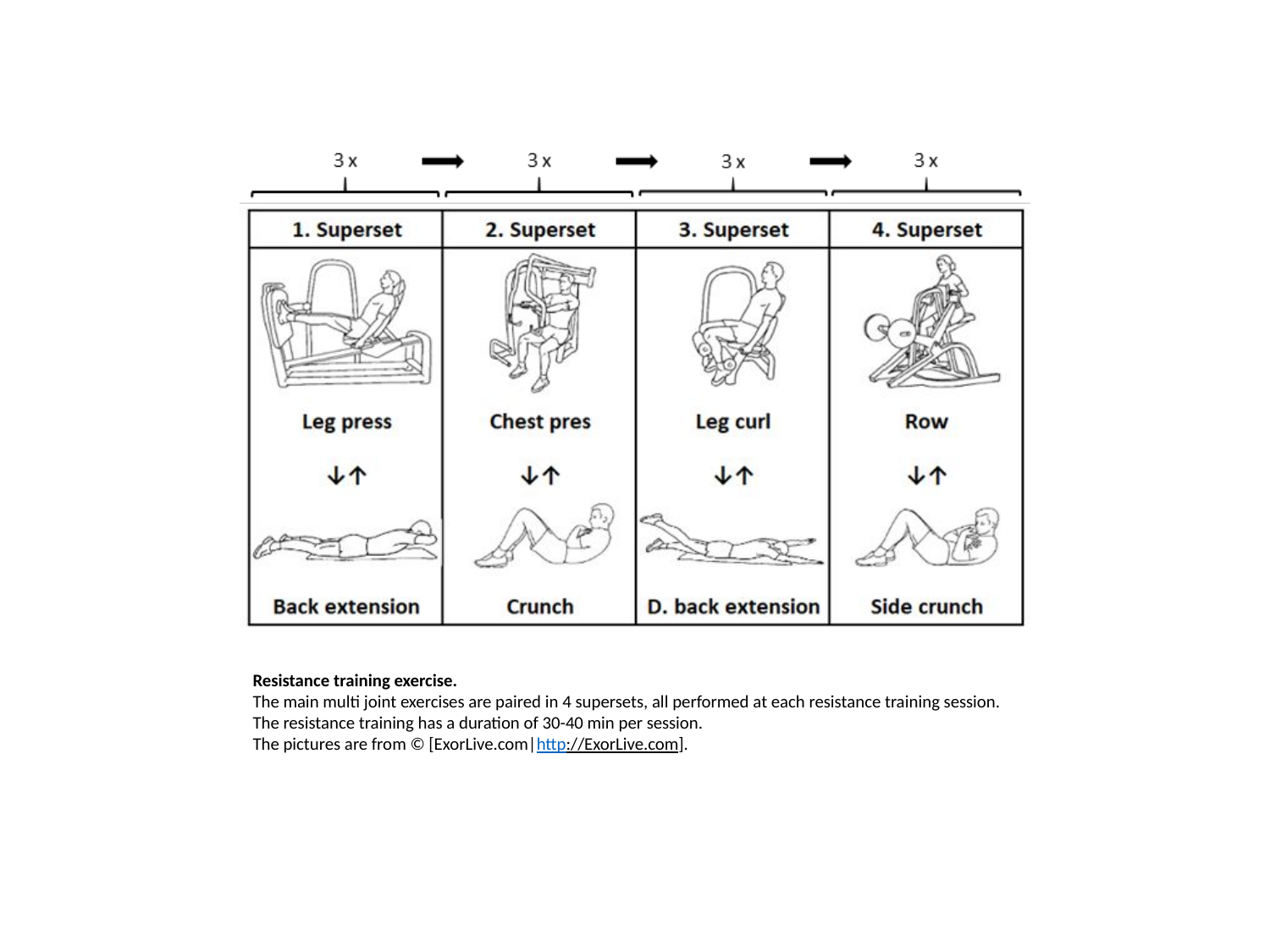

Resistance training exercise.
The main multi joint exercises are paired in 4 supersets, all performed at each resistance training session. The resistance training has a duration of 30-40 min per session.
The pictures are from © [ExorLive.com|http://ExorLive.com].
