# Supplementary material for: The effects of different doses of exercise on pancreatic β-cell function in patients with newly diagnosed type 2 diabetes: study protocol for and rationale behind the “DOSE-EX” multi-arm parallel-group randomised clinical trial
Source: Trials. 2021 Apr 1;22:244. doi: 10.1186/s13063-021-05207-7 (PMC8017660; doi:10.1186/s13063-021-05207-7)
Supplement: Supplementary file 2 — Additional file 2. Algorithm for pharmacological management. [file 13063_2021_5207_MOESM2_ESM.pptx]

## Slide 1
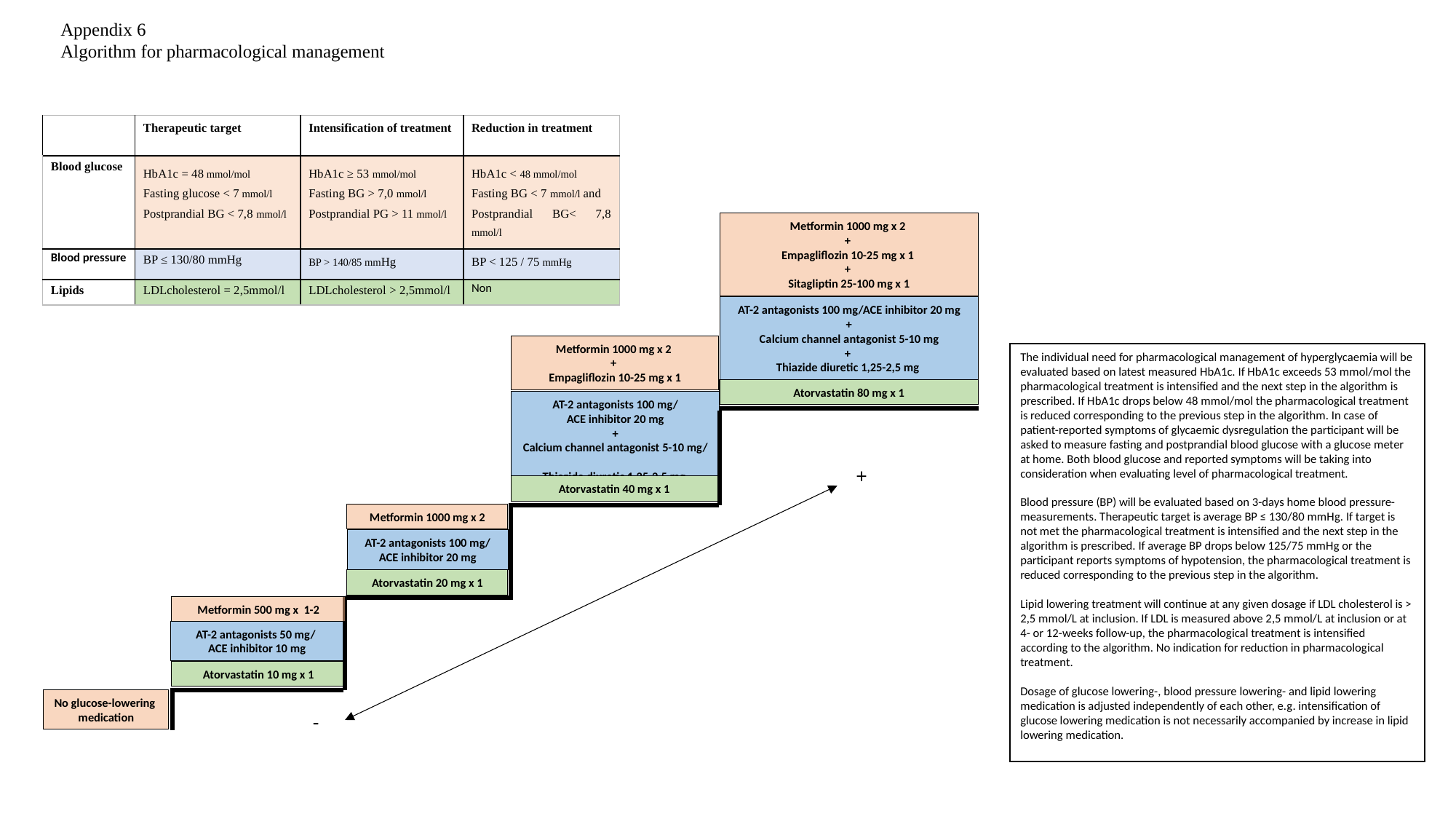

Appendix 6
Algorithm for pharmacological management
| | Therapeutic target | Intensification of treatment | Reduction in treatment |
| --- | --- | --- | --- |
| Blood glucose | HbA1c = 48 mmol/mol Fasting glucose < 7 mmol/l Postprandial BG < 7,8 mmol/l | HbA1c ≥ 53 mmol/mol Fasting BG > 7,0 mmol/l Postprandial PG > 11 mmol/l | HbA1c < 48 mmol/mol Fasting BG < 7 mmol/l and Postprandial BG< 7,8 mmol/l |
| Blood pressure | BP ≤ 130/80 mmHg | BP > 140/85 mmHg | BP < 125 / 75 mmHg |
| Lipids | LDLcholesterol = 2,5mmol/l | LDLcholesterol > 2,5mmol/l | Non |
Metformin 1000 mg x 2
+
Empagliflozin 10-25 mg x 1
+
Sitagliptin 25-100 mg x 1
AT-2 antagonists 100 mg/ACE inhibitor 20 mg
+
Calcium channel antagonist 5-10 mg
+
Thiazide diuretic 1,25-2,5 mg
Metformin 1000 mg x 2
+
Empagliflozin 10-25 mg x 1
The individual need for pharmacological management of hyperglycaemia will be evaluated based on latest measured HbA1c. If HbA1c exceeds 53 mmol/mol the pharmacological treatment is intensified and the next step in the algorithm is prescribed. If HbA1c drops below 48 mmol/mol the pharmacological treatment is reduced corresponding to the previous step in the algorithm. In case of patient-reported symptoms of glycaemic dysregulation the participant will be asked to measure fasting and postprandial blood glucose with a glucose meter at home. Both blood glucose and reported symptoms will be taking into consideration when evaluating level of pharmacological treatment.
Blood pressure (BP) will be evaluated based on 3-days home blood pressure-measurements. Therapeutic target is average BP ≤ 130/80 mmHg. If target is not met the pharmacological treatment is intensified and the next step in the algorithm is prescribed. If average BP drops below 125/75 mmHg or the participant reports symptoms of hypotension, the pharmacological treatment is reduced corresponding to the previous step in the algorithm.
Lipid lowering treatment will continue at any given dosage if LDL cholesterol is ˃ 2,5 mmol/L at inclusion. If LDL is measured above 2,5 mmol/L at inclusion or at 4- or 12-weeks follow-up, the pharmacological treatment is intensified according to the algorithm. No indication for reduction in pharmacological treatment.
Dosage of glucose lowering-, blood pressure lowering- and lipid lowering medication is adjusted independently of each other, e.g. intensification of glucose lowering medication is not necessarily accompanied by increase in lipid lowering medication.
Atorvastatin 80 mg x 1
AT-2 antagonists 100 mg/
ACE inhibitor 20 mg
+
Calcium channel antagonist 5-10 mg/
Thiazide diuretic 1,25-2,5 mg
+
Atorvastatin 40 mg x 1
Metformin 1000 mg x 2
AT-2 antagonists 100 mg/
ACE inhibitor 20 mg
Atorvastatin 20 mg x 1
Metformin 500 mg x 1-2
AT-2 antagonists 50 mg/
ACE inhibitor 10 mg
Atorvastatin 10 mg x 1
No glucose-lowering
medication
-
