# Supplementary material for: The effects of different doses of exercise on pancreatic β-cell function in patients with newly diagnosed type 2 diabetes: study protocol for and rationale behind the “DOSE-EX” multi-arm parallel-group randomised clinical trial
Source: Trials. 2021 Apr 1;22:244. doi: 10.1186/s13063-021-05207-7 (PMC8017660; doi:10.1186/s13063-021-05207-7)
Supplement: Supplementary file 3 — Additional file 3. Outcome Table Article 2. [file 13063_2021_5207_MOESM3_ESM.docx]

| **Outcome** | **Time frame** | **Domain** | **Measurements** |
| --- | --- | --- | --- |
| Other | Base line, 4 weeks, 12 weeks and after 16 weeks | Systemic oxidative stress | Urinary markers of systemic oxidative stress   - 8-oxo-7,8-dihydroguanosine (8-oxo-Guo) Systemic marker of RNA oxidation - 8-Oxo-2'-deoxyguanosine (8-oxo-dG) Systemic marker of DNA oxidation |
| Other | Baseline and after 16 weeks | Glycaemic variability | Continuous glucose monitoring (CGM)   - %Time in range %High %Low - Coefficient of variance (CV) - Standard deviation (SD) - Mean amplitude of glycaemic excursions (MAGE) |
| Other | Base line, 4 weeks, 12 weeks and after 16 weeks | Markers of glycation | - Advanced glycation end-products (AGEs) - Receptors for Advanced glycation end-products |
| Other | Base line, 4 weeks, 12 weeks and after 16 weeks | Systemic low-grade inflammation | Circulating inflammatory markers   - High-sensitive C-reactive protein (hCRP) - Interferon-ϒ, - IL-10, IL-8, IL-6, IL1, - TNFα |
| Other | Baseline and after 16 weeks | Physical fitness | - - Maximal aerobic capacity (VO_2_ peak)   - One-repetition maximum (RM) strength |
| Other | Base line, 4 weeks, 12 weeks and after 16 weeks | Blood glucose control | - HbA1c - Fasting glucose - Fasting C-peptid and insulin |
| Other | Base line, 4 weeks, 12 weeks and after 16 weeks | Blood lipids | - Total cholesterol - Triglyceride - Low- and high-density lipoprotein |
| Other | Base line, 4 weeks, 12 weeks and after 16 weeks | Blood pressure | - Resting systolic and diastolic blood pressure |

**Article 2 – systemic oxidative stress and development of diabetic complications**
