# Supplementary material for: The effects of different doses of exercise on pancreatic β-cell function in patients with newly diagnosed type 2 diabetes: study protocol for and rationale behind the “DOSE-EX” multi-arm parallel-group randomised clinical trial
Source: Trials. 2021 Apr 1;22:244. doi: 10.1186/s13063-021-05207-7 (PMC8017660; doi:10.1186/s13063-021-05207-7)
Supplement: Supplementary file 5 — Additional file 5. Mixed Meal Tolerance Test. [file 13063_2021_5207_MOESM5_ESM.pptx]

## Slide 1
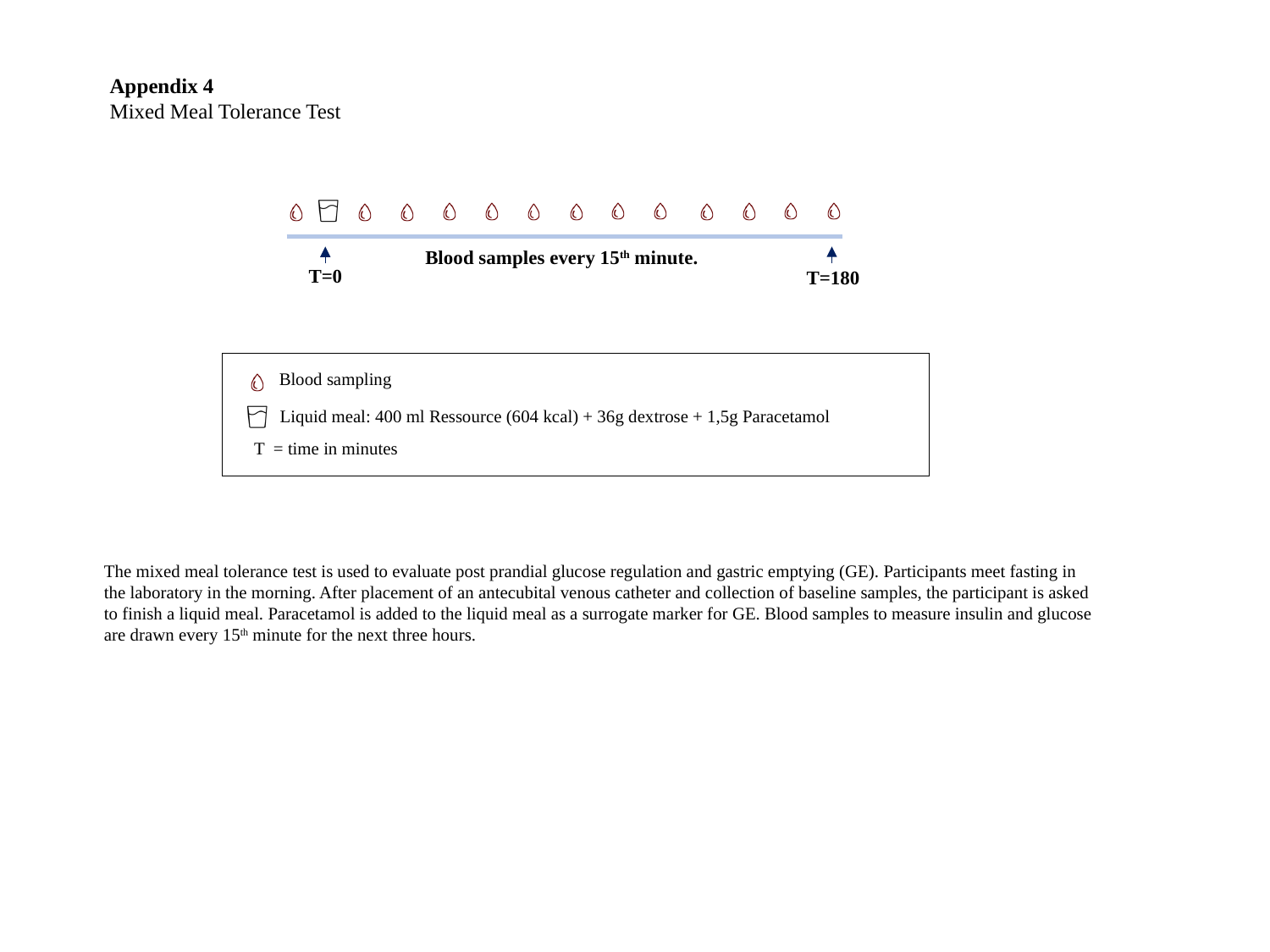

Appendix 4
Mixed Meal Tolerance Test
Blood samples every 15th minute.
T=180
T=0
Blood sampling
Liquid meal: 400 ml Ressource (604 kcal) + 36g dextrose + 1,5g Paracetamol
T = time in minutes
The mixed meal tolerance test is used to evaluate post prandial glucose regulation and gastric emptying (GE). Participants meet fasting in the laboratory in the morning. After placement of an antecubital venous catheter and collection of baseline samples, the participant is asked to finish a liquid meal. Paracetamol is added to the liquid meal as a surrogate marker for GE. Blood samples to measure insulin and glucose are drawn every 15th minute for the next three hours.
