# Supplementary material for: The effects of different doses of exercise on pancreatic β-cell function in patients with newly diagnosed type 2 diabetes: study protocol for and rationale behind the “DOSE-EX” multi-arm parallel-group randomised clinical trial
Source: Trials. 2021 Apr 1;22:244. doi: 10.1186/s13063-021-05207-7 (PMC8017660; doi:10.1186/s13063-021-05207-7)
Supplement: Supplementary file 6 — Additional file 6. Suspension Note. [file 13063_2021_5207_MOESM6_ESM.pdf]

Copenhagen
March 13th 2020

DOSE-EX study group response to the COVID-19 suspension of the public sector in Denmark

Due to the suspension, imposed by the Danish government *, of activities not related to health care in the public sector in Denmark, inclusion of new participants in the DOSE-EX study is suspended until further notice. Activities for included participants, not yet allocated, has been postponed until further notice.

On behalf of the steering committee

Mathias Ried-Larsen

Mathias Ried-Larsen, PhD.
Senior researcher and group leader
Prinicipal investigator DOSE-EX
Centre for Physical Activity Research
Copenhagen University Hospital (Rigshospitalet)
Denmark
E-mail: Mathias.ried-larsen@regionh.dk

*https://www.thelocal.dk/20200311/denmark-to-shut-all-schools-and-universities-to-fight-virus (entry: March 13th 2020 09.00 AM)

CENTRE FOR PHYSICAL ACTIVITY RESEARCH
