# Supplementary material for: The effects of different doses of exercise on pancreatic β-cell function in patients with newly diagnosed type 2 diabetes: study protocol for and rationale behind the “DOSE-EX” multi-arm parallel-group randomised clinical trial
Source: Trials. 2021 Apr 1;22:244. doi: 10.1186/s13063-021-05207-7 (PMC8017660; doi:10.1186/s13063-021-05207-7)
Supplement: Supplementary file 7 — Additional file 7. Informed Consent. [file 13063_2021_5207_MOESM7_ESM.pdf]

DET VIDENSKABSETISKE KOMITÉSYSTEM

(S4)

Informeret samtykke til deltagelse i et sundhedsvidenskabeligt forskningsprojekt.

Forskningsprojektets titel: The effects of different doses of exercise on pancreatic β-cell function in patients with newly diagnosed type 2 diabetes (DOSE-EX): A randomized clinical trial

Erklæring fra forsøgspersonen:

Jeg har fået skriftlig og mundtlig information og jeg ved nok om formål, metode, fordele og ulemper til at sige ja til at deltage.

Jeg ved, at det er frivilligt at deltage, og at jeg altid kan trække mit samtykke tilbage uden at miste mine nuværende eller fremtidige rettigheder til behandling.

Jeg giver samtykke til, at deltage i forskningsprojektet og til, at mit biologiske materiale udtages med henblik på opbevaring i en forskningsbiobank. Jeg har fået en kopi af dette samtykkeark samt en kopi af den skriftlige information om projektet til eget brug.

Forsøgspersonens navn: ____

Dato: _______________ Underskrift: ____

Hvis der kommer nye væsentlige helbredsoplysninger frem om dig i forskningsprojektet vil du blive informeret. Vil du **frabede** dig information om nye væsentlige helbredsoplysninger, som kommer frem i forskningsprojektet, bedes du markere her: __________ (sæt x)

Ønsker du at blive informeret om forskningsprojektets resultat samt eventuelle konsekvenser for dig?:

Ja _____ (sæt x)   Nej _____ (sæt x)

Erklæring fra den, der afgiver information:

Jeg erklærer, at forsøgspersonen har modtaget mundtlig og skriftlig information om forsøget.

Efter min overbevisning er der givet tilstrækkelig information til, at der kan træffes beslutning om deltagelse i forsøget.

Navnet på den, der har afgivet information:

Dato: _______________ Underskrift: ____

Projektidentifikation: (Fx komiteens Projekt-ID, EudraCT nr., versions nr./dato eller lign.)

Samtykkeerklæring, ver. 2, 22/10/18
Anmeldelses nr: 64560
